# Two-dimensional NMR from a single pulse: Reconstructing heteronuclear 2D spectra via off-resonance decoupling and deep neural networks

**DOI:** 10.1073/pnas.2527937123

**Published:** 2026-04-21

**Authors:** Nihar P. Khandave, Veera Mohana Rao Kakita, Charles J. Buchanan, Vaibhav Kumar Shukla, Kevin Haubrich, Pramodh Vallurupalli, D. Flemming Hansen

**Affiliations:** ^a^Tata Institute of Fundamental Research, Gopanpally Village, Serilingampally Mandal, Ranga Reddy District, Hyderabad, Telangana 500107, India; ^b^Department of Structural and Molecular Biology, Division of Biosciences, University College London, London WC1E 6BT, United Kingdom; ^c^The Francis Crick Institute, London NW1 1AT, United Kingdom; ^d^Evolutionary Biochemistry Group, Max Planck Institute for Terrestrial Microbiology, Marburg 35043, Germany; ^e^Department of Biology, Marburg University, Marburg 35043, Germany

**Keywords:** multi-dimensional NMR, methyl groups, deep learning, large proteins, single-pulse NMR

## Abstract

NMR spectroscopy is ubiquitous in many areas of science, including chemistry, material science, biophysics, and structural biology. Heteronuclear two-dimensional (2D) NMR experiments form the basis of several NMR-based investigations, especially those involving biomolecules like proteins. However, in solution, as the molecule of interest becomes larger, transverse relaxation times of the spins of interest become shorter. This makes it difficult to record 2D ^1^H–^13^C or ^1^H–^15^N correlation maps of large protein molecules using standard Fourier transform–based experiments as they contain transfer delays that are long compared to the short relaxation times. Herein, we explore the possibility of leveraging deep neural networks (DNNs) to obtain 2D correlation maps of proteins while eliminating transfer delays from experiments. In this study, we show that 2D methyl ^1^H–^13^C correlation maps can be obtained from experiments containing only a single ^1^H excitation pulse followed by off-resonance ^13^C continuous-wave decoupling. A DNN was successfully trained to reconstruct the 2D ^1^H–^13^C correlation map from datasets recorded with two ^13^C decoupling fields using protein samples enriched with isoleucine, leucine, and valine (ILV) ^13^CHD_2_ methyl-groups in a ^2^H background. The efficacy of this strategy is demonstrated by using the DNN to reconstruct methyl ^1^H–^13^C correlation maps of the ~8 kDa FF domain from human HYPA/FBP11, the ~18 kDa T4 phage lysozyme, the ~360 kDa α_7_α_7_ particle from the *Thermoplasma acidophilum* proteasome and a ~530 kDa ancestrally reconstructed L_8_S_8_ Rubisco complex. This study illustrates the potential for improving the sensitivity and resolution of NMR spectra by designing experiments tailored for use with DNNs.

## Significance

Multidimensional heteronuclear solution state NMR spectroscopy underpins studies of protein dynamics and interactions. However, the experiments have thus far required magnetization-transfer periods and evolution delays, both of which rapidly erode the signal arising from large biomolecules. We introduce here a single-pulse strategy wherein these delays are replaced by off-resonance continuous-wave decoupling so that the two-dimensional correlation map can be reconstructed from the off-resonance decoupling data using a deep neural network. The approach yields high-quality methyl correlation spectra for proteins spanning ~8 to 530 kDa. The codesign of NMR experiments along with deep neural networks establishes a general framework to acquire multidimensional NMR spectra using nontraditional strategies that can be extended to other spin systems and higher-dimensional experiments.

NMR spectroscopy is an important tool among others in material science, chemistry, and structural biology largely due to the development of Fourier-transform (FT) multidimensional NMR experiments (1). In particular, heteronuclear multidimensional NMR experiments have impacted the study of biomolecular structure and dynamics in solution (2–7). Two-dimensional (2D) ^1^H–^15^N and ^1^H–^13^C correlation maps are now routinely used to inform on a variety of processes, such as conformational dynamics by measuring relaxation properties of nuclei, or biomolecular interactions by monitoring peak positions and linewidths as a function of ligand concentration, etc. (4). The heteronuclear 2D correlation experiments also form the primary building block for most higher-dimensional NMR experiments (4, 7).

The key ingredients of the powerful heteronuclear multidimensional experiments are pulse-sequence blocks that transfer magnetization between adjacent nuclei via ^1^ *J* scalar couplings and chemical shift labeling delays during which the magnetization of interest evolves at the chemical shift of the nucleus being studied (4, 7–9). However, in solution, as the molecules become larger (≳100 kDa), transverse relaxation times become shorter, thus leading to both broader peaks as well as reduced signal intensities due to losses during the transfer periods, making it difficult to record (even) 2D heteronuclear correlation maps with adequate sensitivity and resolution (4, 10, 11). Hence, different NMR experiments are now being developed to record heteronuclear correlation maps from samples that contain large biomolecules. The strategies that these new experiments employ generally entail i) increasing the transverse relaxation time by evolving coherences with favorable relaxation properties (10–12) or by reducing the ^1^H density via ^2^H enrichment (13) and ii) reducing the length of experiments by reducing the number of the delays in the experiments (14–16) or by utilizing site-specific isotope labeling strategies that isolates the spins of interest by eliminating ^1^*J*/^2^*J* coupling partners thus avoiding constant time (CT) delays in experiments (17).

An upcoming and promising strategy to improve the quality of NMR spectra involves using deep neural networks (DNNs) in combination with new experimental designs, because it is now becoming clear that DNNs can be trained to distil the key information from complex information-rich NMR data (18–23). In particular, synergistic approaches that combine the development of NMR pulse-sequences and DNNs are beginning to substantially impact biomolecular NMR. Examples include DNNs that virtually decouple and enhance the resolution of methyl and aromatic ^1^H–^13^C correlation maps acquired on uniformly ^13^C labeled protein samples from relatively large proteins (methyl ~360 kDa, aromatic ~50 kDa) that could not be obtained using standard CT approaches (24, 25). Aromatic groups in proteins contain ^13^C sites bonded to varying numbers of other ^13^C nuclei leading to singlets, doublets, and triplets in the ^13^C dimension of a regular ^1^H–^13^C correlation map. Hence, additional information is required to virtually decouple a regular aromatic ^1^H–^13^C spectrum into a resolution-enhanced “decoupled” ^1^H–^13^C correlation map that contains only singlets. Providing additional information (along a pseudo-dimension) obtained using a new experiment that encodes the multiplet information allowed the DNN to reconstruct high-resolution decoupled ^1^H–^13^C correlation maps, thereby illustrating the power of synergistically developing new NMR experiments along with DNNs (25). Key to the success of this strategy is that additional information is provided (in a pseudo-dimension) to the DNN and that (short) non-CT experiments are recorded, instead of the traditional CT experiments, that are often long compared to the transverse relaxation times of the ^13^C nuclei being studied.

**Fig. 1.**
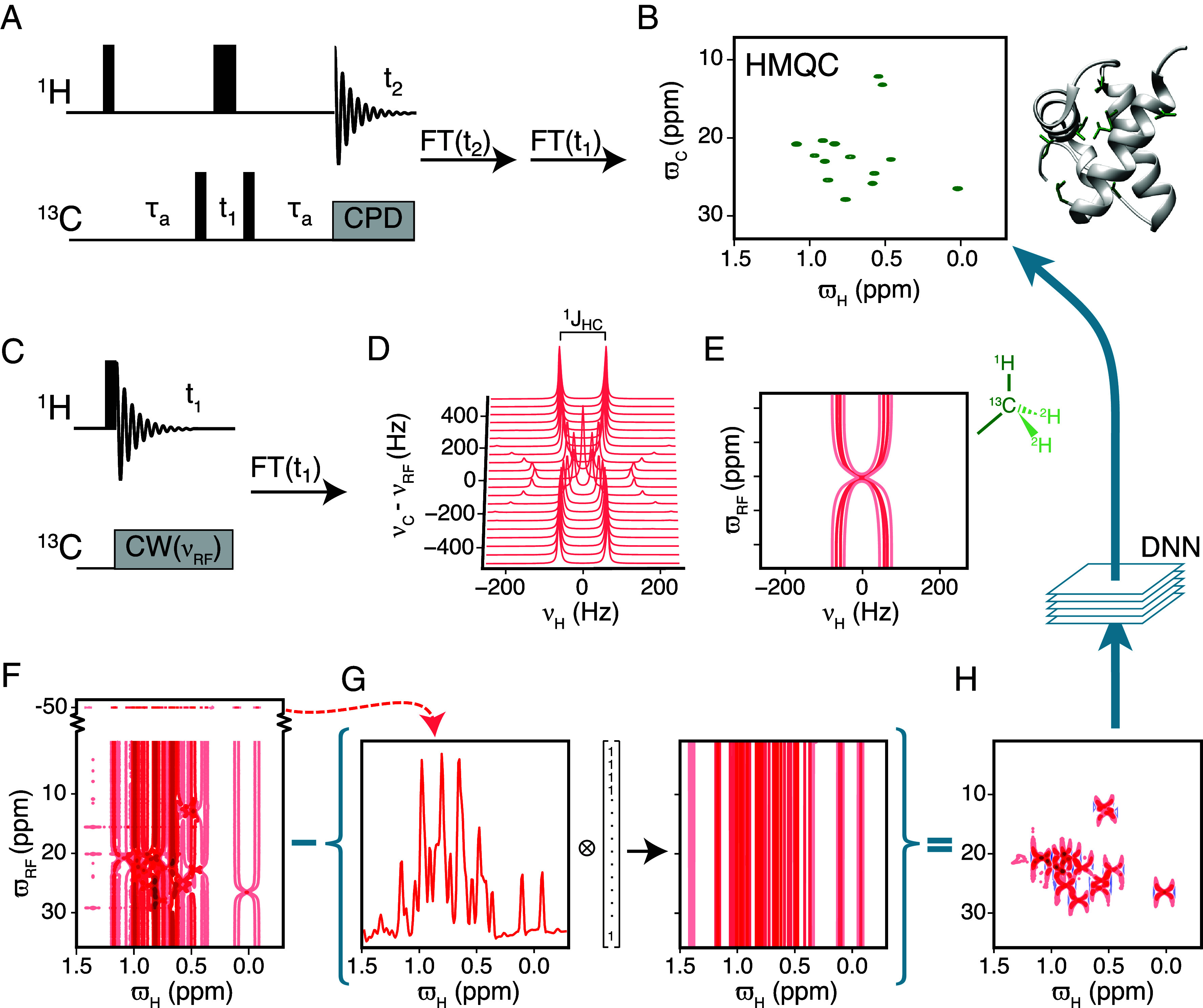
(*A*) The standard ^1^H–^13^C HMQC pulse sequence, which is traditionally used to obtain two-dimensional ^1^H–^13^C correlation spectra *via* 2D Fourier transformation. (*B*) The methyl region of the ^1^H–^13^C HMQC spectrum recorded on a ^13^CHD_2_ ILV isotopically labeled sample of the FF domain, which contains in total fourteen isoleucine, leucine, and valine residues. (*C*) The “single pulse” pulse experiment that forms the basis for the proposed approach to obtain 2D ^1^H–^13^C correlation maps. After the ^1^H excitation pulse, a weak ^13^C CW decoupling is applied at various ^13^C offsets, $\nu_{RF}$, while recording the ^1^H spectrum. Fourier transform along the ^1^H dimension provides an off-resonance dataset as shown in (*D* and *E*) (contour plot). In (*D* and *E*) the off-resonance dataset is calculated using a ^13^C CW decoupling strength of $B_{1}$ (^13^C) = 110 Hz and a one bond ^13^C–^1^H scalar coupling of ^1^*J*_HC_ = 125 Hz. The resonance frequencies of the ^1^H and ^13^C nuclei are set to 0 Hz. All the transverse relaxation rates were set to 25 s^−1^. (*F*) ^1^H–^13^C off-resonance dataset recorded on the FF domain, where substantial overlap is clear. (*G*) The 1D ^1^H reference spectrum of the FF domain, obtained with far off-resonance ^13^C decoupling, −50 ppm, contains doublets and solvent signals. (*H*) The ^1^H–^13^C (difference) off-resonance dataset obtained by subtracting the (coupled) ^1^H 1D reference spectrum in (*G*) from the ^1^H spectrum in (*F*). Overlap is reduced in the (difference) off-resonance dataset (*F* vs. *H*) and can be transformed by a DNN into a standard ^1^H–^13^C correlation map. All spectra of the FF domain were recorded on a 16.4 T (700 MHz) spectrometer at 6.5 °C.

With the ultimate goal of recording heteronuclear 2D correlation maps of large macromolecular systems (preferably uniformly labeled), we sought to decrease the length of the pulse sequences even further than what has been done previously (14, 15). We therefore revisited the idea of using a single excitation pulse followed by off-resonance decoupling to correlate spins that are scalar coupled to one another (26–29). Specifically, herein we explore the possibility of developing a DNN to generate a regular high-resolution 2D ^1^H–^13^C correlation map from these customized datasets recorded on an isolated ^1^H–^13^C spin-system using an experiment with a single ^1^H excitation pulse (Fig. 1 and *SI Appendix*, Fig. S1). In this proof of principle study, we find that a simple DNN can transform protein methyl (^13^CHD_2_) off-resonance data into a methyl ^1^H–^13^C correlation map. We demonstrate the applicability of the single pulse method by reconstructing the methyl ^1^H–^13^C correlation maps of four different protein constructs, that is, the FF domain (30) from human HYPA/FBP11 (FF domain, ~8 kDa), T4 phage lysozyme (T4L, ~18 kDa) (31), the double-α-ring particle of the *Thermoplasma acidophilum* proteasome ($\alpha_{7}\alpha_{7}$, ~360 kDa) (32) and a hexadecameric ancestrally reconstructed Rubisco (L_8_S_8_, ~530 kDa) that contains eight copies each of the ~13 kDa small (S) and ~54 kDa large (L) subunits (33).

## Results

Over the last couple of decades, it has become clear that methyl groups are sensitive reporters of the structure and dynamics of large proteins in solution. Consequently, several methyl NMR experiments and labeling strategies have been developed to study protein structure and dynamics (17). Concomitant with the discovery of the methyl-TROSY effect it was shown that methyl ^1^H–^13^C correlation maps with high S/N can be recorded using the simple four-pulse (^1^H–^13^C) HMQC experiment (34) (Fig. 1 *A* and *B*) that exploits the methyl-TROSY effect (10). The HMQC experiment consists of two ^1^H↔^13^C transfer-periods (^1^H→^13^C and ^13^C→^1^H) and a ^13^C evolution period in addition to the ^1^H detection period (Fig. 1*A*). About two decades later, it was shown that an experiment in which the ^13^C→^1^H transfer-period was eliminated by subsuming it into the ^1^H detection period provided spectra with substantially higher sensitivity for large proteins (14, 15). Datasets acquired using this experiment, referred to as delayed-decoupling HMQC (ddHMQC), are processed by the addition of a simple apodization prior to classical discrete Fourier transform. A natural step to attempt to obtain even higher sensitivity is to eliminate the last remaining transfer period (^1^H→^13^C) and consequently the ^13^C evolution period, which leads to the single-pulse experiment, Fig. 1*C*. The extraction of ^13^C indirect frequencies from such experiments has been established (26, 27) as long as there is no overlap of the resulting “patterns.” As detailed below, reconstructing ^1^H–^13^C correlation maps for large proteins with substantial overlap from such datasets is only possible due to the recent developments in machine learning and deep learning. It is therefore now very timely to explore potential opportunities provided by the single-pulse experiment for characterizing large proteins.

### Brief Overview of the Single-Pulse Method.

In the single-pulse experiment (Fig. 1*C*), following the single ^1^H (π/2) excitation pulse, the ^1^H free induction decay (FID) is recorded while applying ^13^C constant-wave (CW) decoupling (35, 36). The one-dimensional (1D) ^1^H frequency-domain spectrum is obtained by Fourier transform of the FID and the process is repeated using different carrier offsets, $\nu_{RF}$, for the ^13^C CW decoupling. Fig. 1*D* illustrates the variation in the ^1^H spectrum as a function of the ^13^C carrier offset relative to the ^13^C carbon resonance frequency ($\nu_{C}$), $\nu_{C}-\nu_{RF}$. In Fig. 1*D* $\nu_{C}$ = 0 Hz, the one bond ^1^H–^13^C coupling constant ^1^*J*_HC_ is 125 Hz and the ^13^C CW field is applied with a field strength of $B_{1}$ = 110 Hz at various offsets, $\nu_{RF}$, ranging from −500 Hz to +500 Hz in steps of 50 Hz. A contour plot of the spectrum in Fig. 1*D* is shown in Fig. 1*E*, where an X shaped pattern is clearly visible. Overall, when $B_{1}$ is large compared to ^1^*J*_HC_/2, the ^1^H spectrum is approximately a doublet with an effective splitting of J1HC|νC−νRF|/(νC−νRF)2+B12 (4, 36), where $B_{1}$ is the strength of the $B_{1}$ field in Hz. It therefore depends on both the field strength of the ^13^C CW field, $B_{1}$, as well as the carrier offset $\nu_{RF}$. Hence, the ^1^H spectrum consists of a doublet with peaks separated by ^1^*J*_HC_, when the ^13^C CW decoupling is carried out far from the resonance frequency of the ^13^C nucleus in question ($\left|\nu_{C}-\nu_{RF}\right|\gg B_{1}$). On the other hand, when the carrier of the CW decoupling moves closer to the resonance frequency of the ^13^C spin, and $\left|\nu_{C}-\nu_{RF}\right|$ is decreased, the separation between the two peaks of the ^1^H doublet becomes smaller and the doublet collapses to a singlet when $\left|\nu_{C}-\nu_{RF}\right|$ approaches 0 Hz, giving rise to a unique X-shaped pattern centered at the ^13^C and ^1^H chemical shifts (Fig. 1*E*). Of particular interest here is that the series of ^1^H spectra recorded with ^13^C CW decoupling at different offsets, referred to here as an off-resonance dataset, contains information about the chemical shift of both the ^1^H ($\varpi_{H}$; ppm) and the ^13^C ($\varpi_{C}$; ppm) nuclei. In the past, off-resonance decoupling was used to correlate nuclei connected by ^1^ *J* couplings, but these original studies were confined to small molecules due to spectral overlap (26–29, 37). As we aim to ultimately probe large proteins in solution, below we describe some of the strategies that we have used to alleviate the spectral overlap problem by leveraging recent advances in deep learning.

### Applying the Single-Pulse Method to Protein Samples.

Single-pulse off-resonance datasets of even small proteins lead to substantial overlap. The methyl ILV ^1^H–^13^C (HMQC) correlation map recorded using a sample of ^13^CHD_2_ enriched FF domain (~8 kDa) contains a distinct peak for each of the 14 methyl sites, Fig. 1*B*. The methyl ILV ^1^H–^13^C off-resonance dataset recorded on the same sample with $B_{1}$ = 110 Hz is shown in Fig. 1*F*. As expected from the calculations, the characteristic X-shaped patterns are observed, however, the off-resonance dataset is highly overlapped. One reason for the overlap in the off-resonance dataset are the streaks along the ^13^C dimension that arise due to the doublets separated by ^1^*J*_HC_. As mentioned earlier, these doublets arise when the ^13^C CW decoupling is carried out at an offset that is far from the resonance frequency of the ^13^C nucleus. To reduce overlap, a ^1^H 1D reference spectrum is recorded with far off-resonance ^13^C decoupling (−50 ppm; Fig. 1 *F*, *Top*), which is subtracted from every off-resonance 1D spectrum, Fig. 1*G*. The reference 1D spectrum contains ^1^H doublets separated by ^1^*J*_HC_ and thus subtracting it from the off-resonance spectrum eliminates the streaks (Fig. 1*H*) and reduces the overlap (Fig. 1*F* vs. Fig. 1*H*). Additionally, this subtraction also improves the baseline since strong solvent/buffer peaks, etc. that are not affected by the ^13^C decoupling are also removed. A ^1^H spectrum recorded without ^13^C decoupling can also serve as the 1D reference spectrum. The off-resonance spectra from which the reference 1D spectrum has been subtracted will sometimes be referred to as difference off-resonance spectra.

Deep learning is renowned for extracting, simplifying, and transforming features in complex datasets and has been extensively used to simplify and enhance NMR spectra (20, 38). Here, we use a DNN to transform single-pulse off-resonance datasets (from protein samples) that contain the unique X-shaped patterns (features) into classical 2D NMR correlation maps with cross-peaks at the appropriate positions. This effectively means that a standard methyl 2D (^1^H–^13^C) correlation map can be obtained using the single-pulse experiment that does not contain any ^1^H↔^13^C transfer periods or a ^13^C evolution period. In this proof of principle study, we only focused on ^13^CHD_2_ methyl groups because i) the methyl region of a protein’s ^1^H–^13^C NMR spectrum contains peaks that originate only from methyl groups unlike, for example, the amide region that contain both ^15^NH and ^15^NH_2_ resonances, ii) well-established protocols are available to enrich proteins with isolated methyl groups within a silent deuterated background (17, 39), and iii) to efficiently generate synthetic training data for the DNN one can treat the ^13^CHD_2_ groups as isolated two-spin (^1^H–^13^C) systems. To further aid the DNN in robustly transforming single-pulse off-resonance data, as discussed above, we provide the DNN with an additional pseudo-dimension consisting of two different off-resonance datasets, one with a $B_{1}$ of 110 Hz and one with a $B_{1}$ of 220 Hz (*Discussion*). As we show below, this allows for the DNN to transform the two off-resonance spectra into the appropriate ^1^H–^13^C correlation map similar to the ^1^H–^13^C HMQC (or HSQC) (34, 40).

### Developing a DNN to Transform Off-Resonance Datasets into ^1^H–^13^C Correlation Maps.

Training a DNN via supervised learning requires a large amount of training data, where the amount typically relates to the complexity of the neural network architecture (41). As this is a proof-of-principle study, we trained a simple DNN architecture with seven convolutional layers (~4.5 million weights) to carry out the transformation (*Materials and Methods* and *SI Appendix*, Fig. S2) as opposed to training the well-established but more complex FID-Net-2 (25, 42) that provides uncertainty estimates in addition to carrying out the desired transformation. The training data used to train the convolutional DNN consist of difference off-resonance datasets, akin to Fig. 1*H*, and the corresponding target ^1^H–^13^C correlation map, akin to Fig. 1*B*. As a large number of such datasets are not available, and DNNs have in the past been successfully trained to transform experimental magnetic resonance data using synthetic data (23, 43), we too generated synthetic data for training the DNN. More specifically, the difference off-resonance datasets consisted of two $I\left(\varpi_{H},\varpi_{C}\right)$ matrices (512 × 200) calculated for two $B_{1}$ values (~220/110 Hz). The sweepwidth in the ^1^H dimension is ~5 ppm, and the 200 ^13^C decoupling offsets span ~30 ppm (*SI Appendix*, Table S1) covering the entire methyl region of the ^1^H–^13^C correlation map. Synthetic off-resonance datasets were generated by propagating the Bloch equations with a range of parameters appropriate for methyl groups in proteins, including chemical shifts, relaxation rates, ^1^*J*_HC_ couplings, etc. Global parameters, those common to all methyl groups in one protein, include $B_{0}$ (field strength ~16.4 T), $B_{1}$ (^13^C decoupling power, ~220/110 Hz), ^13^C/^1^H sweep widths, etc. were slightly varied while generating the training data (*Materials and Methods*). The target ^1^H–^13^C correlation map was represented by a single $I\left(\varpi_{H},\varpi_{C}\right)$ matrix (512 × 200), with Gaussian-shaped peaks at the appropriate $\varpi_{H}$ and $\varpi_{C}$ chemical shifts. A Gaussian shape was used for the cross-peaks in the target, as opposed to a Lorentzian shape, because of its more localized nature, making it desirable from an NMR perspective. Gaussian shaped peaks are readily obtained by Gaussian apodization in the CT dimension or by using a Lorentzian-to-Gaussian transformation when the signals of interest decay exponentially (4). Although not critical from the NMR perspective, the localized nature of the Gaussian shape might also make it easier to train the DNN to reconstruct the desired target ^1^H–^13^C correlation map from the off-resonance datasets due to less stringent requirements on the receptive fields of the convolutional layers. The linewidths, that is the Full-Width-at-Half-Maximum, in the target ^1^H–^13^C correlation map of peaks in the ^1^H dimension is determined by $R_{2,H}$/π (minimum value of 20 Hz). Linewidths in the ^13^C dimension were fixed to a constant value (as in CT experiments) of 30 Hz, as the off-resonance datasets do not directly provide ^13^C linewidth information (*Materials and Methods* and Eq. **1**). The DNN (*SI Appendix*, Fig. S2) was trained to convert the synthetic input (~220/~110 Hz) difference off-resonance datasets into ^1^H–^13^C correlation maps (*SI Appendix*, Fig. S3) by minimizing the mean square error (MSE) between the DNN reconstructed ^1^H–^13^C correlation map and the desired target ^1^H–^13^C correlation map. For a validation (evaluation) set of 5,000 synthetic datasets, containing a random number of peaks between 1 and 1,000, the MSE between the DNN reconstructed ^1^H–^13^C correlation map and the target ^1^H–^13^C correlation map was ~0.75 compared to an MSE of ~65 when an untrained DNN with random weights was used to reconstruct ^1^H–^13^C correlation maps (*SI Appendix*, Fig. S2*B*). Two examples of how the trained DNN transforms (synthetic) difference off-resonance datasets into ^1^H–^13^C correlation maps are shown in *SI Appendix*, Fig. S3.

### Reconstructing Experimental Protein Methyl ^1^H–^13^C Correlation Maps from Off-Resonance Datasets.

**Fig. 2.**
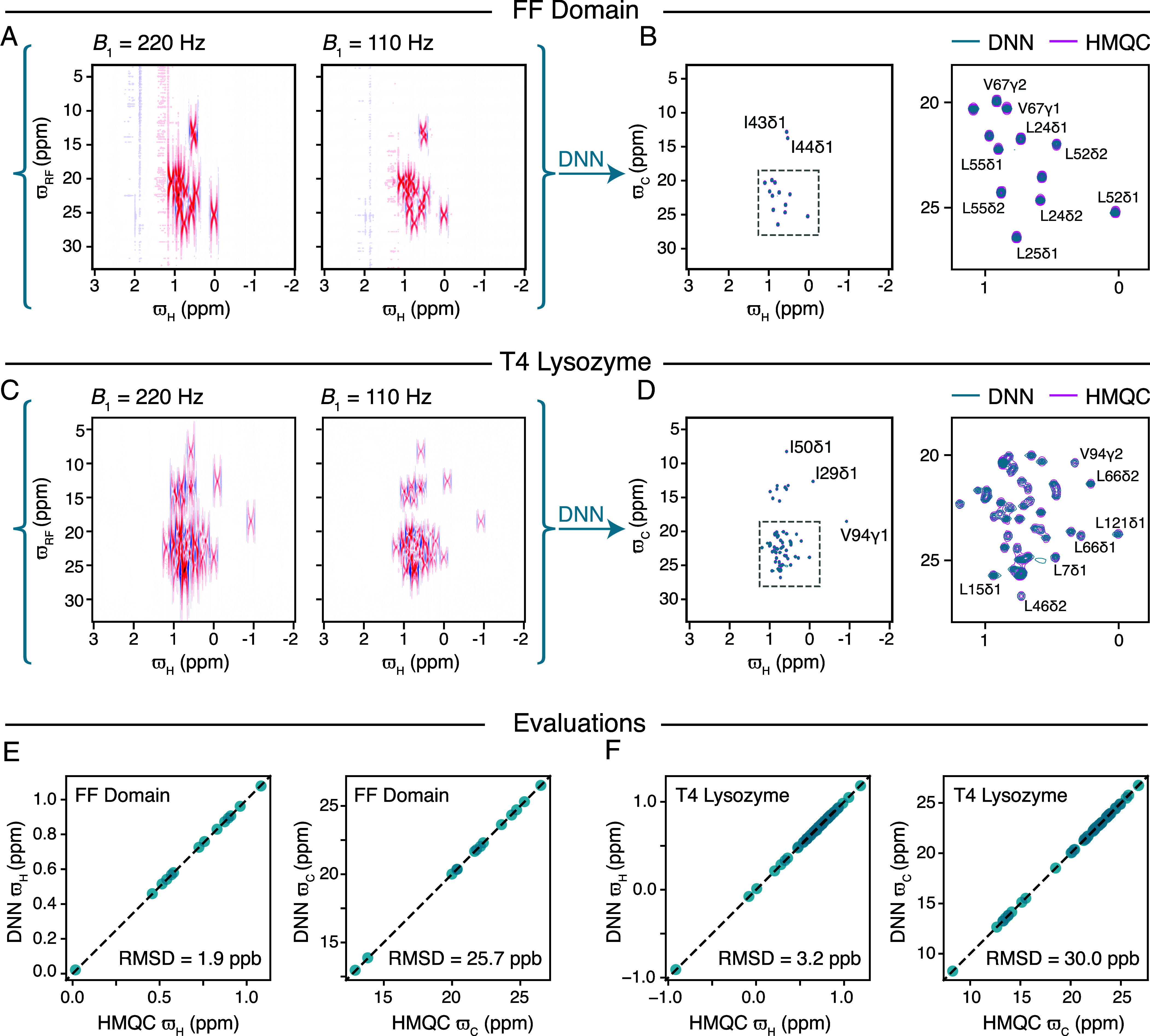
(*A*) Methyl ^1^H–^13^C difference off-resonance dataset for the FF domain with $B_{1}$ (^13^C) = 220 Hz (*Left*) and $B_{1}$ (^13^C) = 110 Hz (*Right*). (*B*) DNN reconstruction of the ^1^H–^13^C correlation map for the FF domain overlaid with the HMQC ^1^H–^13^C correlation map and along with a zoom of the most overlapped region (*Right*). (*C*) Methyl ^1^H–^13^C difference off-resonance dataset for T4 Lysozyme with $B_{1}$ (^13^C) = 220 Hz (*Left*) and $B_{1}$ (^13^C) = 110 Hz (*Right*). (*D*) DNN reconstruction of the ^1^H–^13^C correlation map for T4 Lysozyme overlaid with the HMQC correlation map and along with a zoom of the most overlapped region (*Right*). (*E* and *F*) Evaluation of accuracy of peak positions obtained from the DNN-derived ^1^H–^13^C correlation maps by comparison with peak positions from HMQC correlation spectra, shown for the FF domain (*E*) and T4 Lysozyme (*F*). All the experiments were carried out at 6.5 °C on a 16.4 T (700 MHz) spectrometer.

The trained DNN was first used to transform off-resonance datasets (Fig. 2*A*) recorded on a sample of the ~8 kDa FF domain, which, as mentioned earlier, contains fourteen ^13^CHD_2_ methyl groups. The DNN successfully transformed the off-resonance datasets (Fig. 2*A*) into a ^1^H–^13^C HMQC-like correlation map (Fig. 2*B*) in which peak positions agree well with those in the HMQC. In Fig. 2*B*, the DNN derived ^1^H–^13^C correlation map is shown in cyan while the standard HMQC ^1^H–^13^C map is shown in purple and an excellent agreement is observed. The peak positions along ^1^H and ^13^C, respectively, $\varpi_{H}$ and $\varpi_{C}$, obtained from the DNN derived ^1^H–^13^C correlation map agree well with those derived from the ^1^H–^13^C HMQC with RMSDs of ~2 ppb and ~26 ppb for ^1^H and ^13^C shifts, respectively (Fig. 2*E*). We further proceeded to test the DNN using off-resonance datasets recorded on a sample of the medium sized T4L, which contains 60 (ILV) ^13^CHD_2_ groups (Fig. 2*C*). Although parts of the ^1^H–^13^C map are severely overlapped, the ^1^H–^13^C correlation map generated by the DNN (cyan) is very similar to the HMQC (purple) (Fig. 2*D*) and the chemical shifts of the peaks in the two spectra are again very similar, Fig. 2*F*, with RMSDs of ~3 ppb and ~30 ppb for ^1^H and ^13^C shifts, respectively.

### Assessing the Quantitative Nature of the Transformation.

**Fig. 3.**
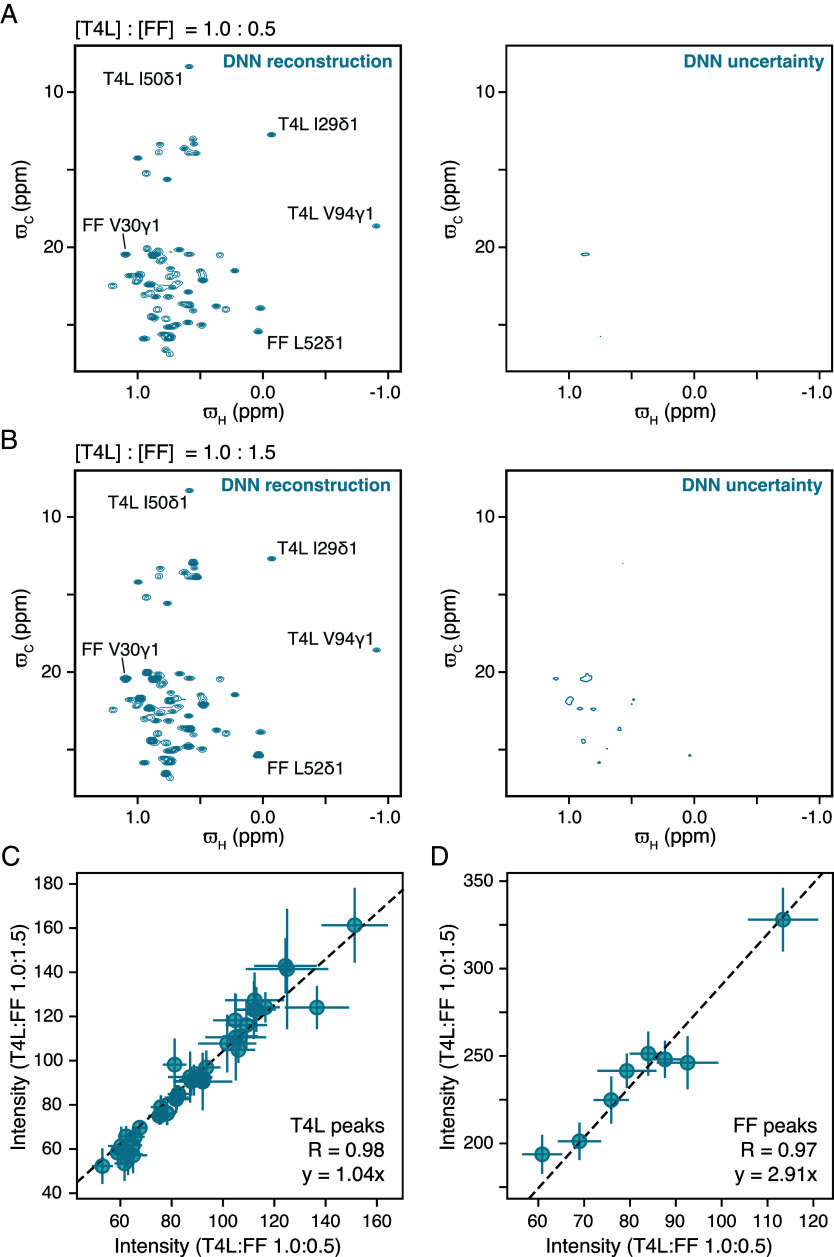
The peak intensities in the DNN reconstructed ^1^H–^13^C map report on concentrations in mixtures of the two proteins T4L and the FF domain. DNN reconstructed ^1^H–^13^C maps and associated intensity errors, when the T4L and FF domain difference off-resonance datasets are coadded in the ratio 1:0.5 (*A*) and 1:1.5 (*B*). Comparison of peak intensities between the DNN derived ^1^H–^13^C maps shown in a and b for resonances arising from T4L (*C*) and FF domain (*D*). Peak intensities were obtained by fitting Gaussian lineshapes (*Materials and Methods* and *SI Appendix*, Fig. S4). Intensities arising from T4L peaks are identical within uncertainty in both the spectra as seen from the correlation in *C*, while the intensity of the peaks arising from the FF domain increases by a factor of 2.91 close to the expected value of 3 (=1.5/0.5). The data used in the analysis presented here is from the experiments depicted in Fig. 2.

Having empirically established that the DNN transforms experimental data acquired using the single-pulse experiment into ^1^H–^13^C correlation maps with cross-peaks at the correct chemical shifts, we next set out to assess the quantitative aspects of the intensities of the cross-peaks in the transformed spectrum. Direct comparison between the intensities of peaks in the DNN generated ^1^H–^13^C correlation map and, for example, a ^1^H–^13^C HMQC/HSQC type spectra will not be meaningful as peak intensities in HSQC and HMQC spectra are sensitive to the ^1^*J*_HC_ couplings, ^13^C/^1^H relaxation rates, and to the exact values of the delays/evolution times used in the experiment. However, it will be meaningful to perform a comparison of relative peak intensities between DNN reconstructed ^1^H–^13^C correlation maps recorded using samples that are mixtures and in which the ratio of the constituents is varied. Thus, we constructed mixtures of the two proteins T4L and FF domain by combining off-resonance datasets from T4L and FF in ratios of 1:0.5 and 1:1.5, respectively (T4L concentration constant), and reconstructing the ^1^H–^13^C correlation maps using the trained DNN. The DNN derived ^1^H–^13^C correlation maps for the two “samples” and their uncertainties are shown in Fig. 3 *A* and *B*, respectively. The uncertainties were estimated by the Monte Carlo dropout method (*SI Appendix*, Figs. S2 and S3 and *Materials and Methods*).

In line with the concentration of T4L being the same in both the samples, peaks that originate from T4L have similar intensities in Fig. 3 *A* and *B*. On the other hand, peaks that originate from the FF domain are more intense in Fig. 3*B* (T4L:FF ratio of 1.0:1.5) than in Fig. 3*A* (T4L:FF ratio of 1.0:0.5) consistent with the concentration of the FF domain being higher in the sample corresponding to Fig. 3*B*. Fig. 3*C* compares the intensities of peaks (*SI Appendix*, Fig. S4) that originate from T4L in the two DNN-derived ^1^H–^13^C correlation maps of the mixtures and, as expected, the intensities of these peaks are similar in the two DNN derived spectra (slope of 1.04). Similarly, Fig. 3*D* compares the intensities of peaks (*SI Appendix*, Fig. S4) that derive from FF in the two DNN derived ^1^H–^13^C correlation maps of the mixtures, and again as expected, the intensities of the FF peaks are higher the sample with ratio of 1:1.5, with a slope of 2.91 compared to the expected slope of 3 (1.5/0.5). Overall, this shows that the intensities of peaks in the ^1^H–^13^C correlation maps reconstructed by the DNN from experimental data are reasonably meaningful. It is possible that modifying the architecture of the DNN and a more informed choice of $B_{1}$ fields and offsets used to record the off-resonance datasets will lead to better estimates of intensities and peak positions.

As the training data for the DNN did not include any artifacts, we wanted to assess the performance of the DNN in the presence of artifacts and thus assess the DNN’s performance on slightly out-of-scope (out-of-distribution) data. Mis-setting the Z1 and Z2 shims on the NMR spectrometer and thus altering the lineshape of the peaks in the input spectra, had almost no effect on the peak positions and also preserved relative peak intensities in the DNN generated FF domain methyl ^1^H–^13^C correlations maps (*SI Appendix*, Fig. S5). While this does not include a full survey of all possible out-of-scope scenarios, it does show that, at least when it comes to lineshape, the DNN can reconstruct spectra under suboptimal conditions that were not included in the training data.

### Reconstructing the Ile ^1^H–^13^C Correlation Map of Large Protein Oligomers.

Having established that the single-pulse experiment, along with the trained DNN, can reconstruct the methyl ^1^H–^13^C correlation maps of small-to-medium sized proteins, we wanted to test the limits of the single-pulse strategy on larger systems with successively lower tumbling times. Thus, we prepared a sample of the ~360 kDa double-α-ring ($\alpha_{7}\alpha_{7}$) of the proteasome that is ^13^CHD_2_ enriched at Ile δ1 positions. Single-pulse experiments were recorded at 50 °C and to make the task more challenging also at 10 °C. At 10 °C the viscosity of D_2_O is about 2.2 times higher than it is at 37 °C and about 2.95 times higher than at 50 °C (44). Thus, the relaxation properties of $\alpha_{7}\alpha_{7}$ at 10 °C are close to that of a ~790 kDa sized protein at 37 °C and a 1 MDa particle at 50 °C.

**Fig. 4.**
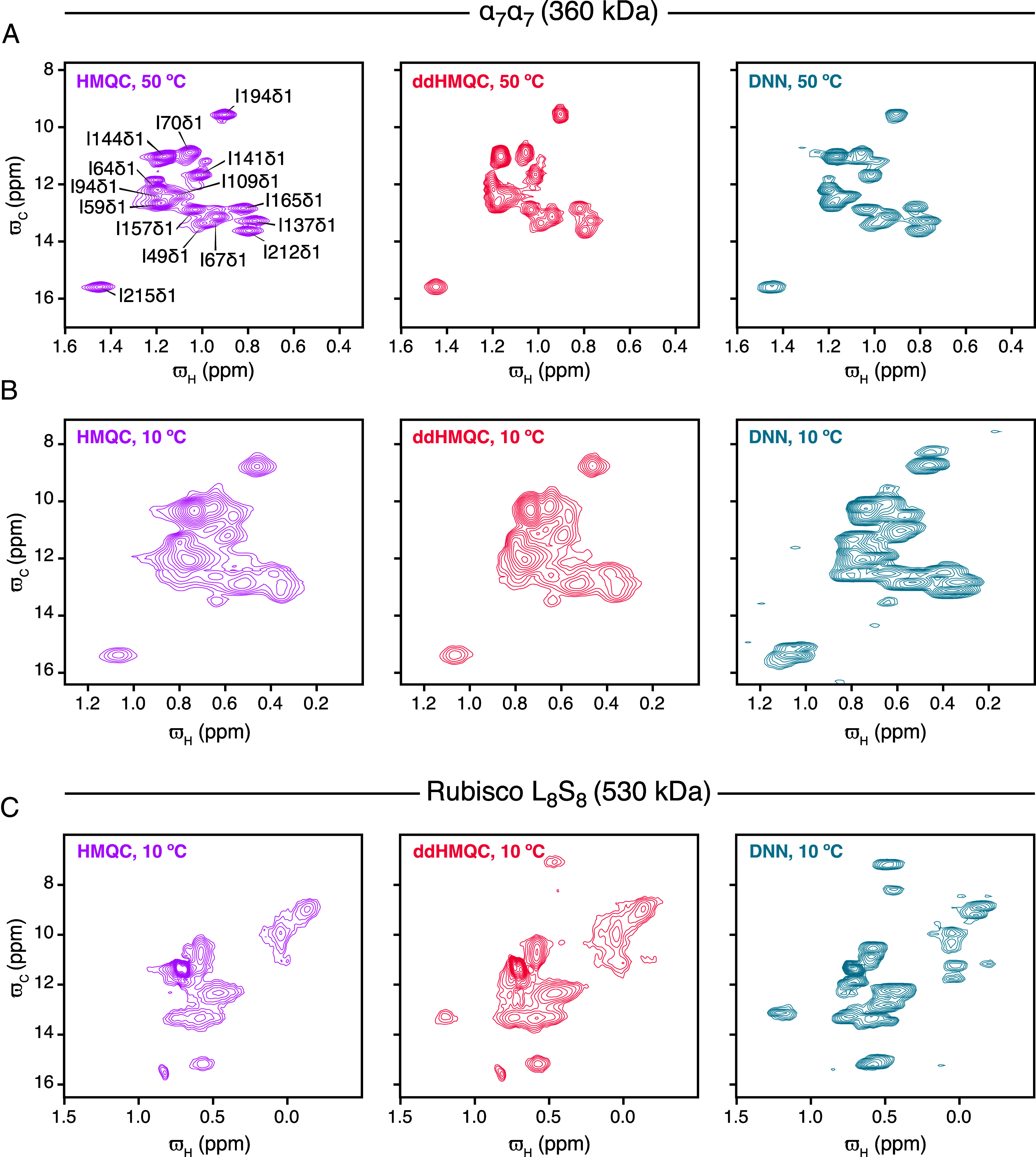
Comparison of the HMQC, ddHMQC and the DNN reconstructed (from off-resonance data) Ile δ1 ^13^C–^1^H correlation maps recorded (16.4 T; 700 MHz) using samples of the $\alpha_{7}\alpha_{7}$ proteasome at 50 °C (*A*), at 10 °C (*B*) and L_8_S_8_ Rubisco at 10 °C (*C*). The $\alpha_{7}\alpha_{7}$ proteasome HMQCs and ddHMQCs (*A* and *B*) were recorded with 32 scans, interscan delay of 1.5 s, acquisition times of 64 ms (^1^H) and 24 ms (^13^C, 48 complex points), and a total acquisition time of about 1.5 h. The L_8_S_8_ Rubisco HMQC and ddHMQC (*C*) were recorded with 156 scans, interscan delay of 2 s, acquisition times of 64 ms (^1^H) and 18.9 ms (^13^C, 100 complex points), and a total acquisition time of about 19 h. In all three cases (above) the single-pulse experiment was recorded in (approximately) the same amount of time as the corresponding HMQC and ddHMQC experiments (*Material and Methods*).

At 50 °C, the single-pulse/DNN derived methyl ^1^H–^13^C correlation map of $\alpha_{7}\alpha_{7}$ is qualitatively of similar quality to the ^1^H–^13^C HMQC spectra and the more sensitive delayed-decoupling ^1^H–^13^C HMQC (Fig. 4*A*). For the substantially more challenging case, $\alpha_{7}\alpha_{7}$ at 10 °C (Fig. 4*B*), cross-peaks that are poorly resolved in the ^1^H–^13^C HMQC/ddHMQC spectra (Fig. 4*B*; central region) are well resolved in the DNN-derived methyl ^1^H–^13^C correlation map (Fig. 4*B*).

To further evaluate the potential of the single-pulse strategy, we prepared a sample of a hexadecameric ancestrally reconstructed ~530 kDa L_8_S_8_ Rubisco in which the L subunit is ^13^CHD_2_ enriched at Ile δ1 positions. The relaxation properties of the ~530 kDa Rubisco at 10 °C are close to that of a ~1.1 MDa particle at 37 °C. As in the case above of $\alpha_{7}\alpha_{7}$ at 10 °C, we find that peaks that are severely overlapped in the HMQC and ddHMQC correlation maps (Fig. 4*C*) are visible and resolved in the spectrum reconstructed by the DNN (Fig. 4*C*). Thus, with these large molecules ($\alpha_{7}\alpha_{7}$ and L_8_S_8_ at 10 °C), the single-pulse/DNN derived correlation maps (Fig. 4 *B* and *C*) appear to be qualitatively better than the HMQC and ddHMQC correlation maps (Fig. 4 *B* and *C*) suggesting that the single-pulse approach can be used to obtain ^1^H–^13^C correlation maps when the HMQC and ddHMQC derived ^1^H–^13^C correlation maps are of poor quality. It is possible that peaks that are broadened beyond detection in HMQC/ddHMQC experiments due to chemical exchange in the ^13^C dimension can be detected by the single-pulse strategy.

The HMQC and ddHMQC are very sensitive experiments for recording ^1^H–^13^C 2D correlation maps from ^13^CHD_2_ methyl groups in a ^2^H background as the ^1^H–^13^C MQ coherence has very favorable relaxation properties because the ^13^C and ^1^H chemical shift anisotropies are small and the ^13^C–^1^H dipolar interactions essentially vanish. However, when evolution in the indirect dimension becomes unfavorable due to short transverse relaxation times, we expect that the single-pulse experiment will outperform traditional FT-based 2D experiments for recording ^1^H–^13^C 2D correlation maps in large systems suggesting that it will be fruitful to explore the utility of the single-pulse strategy to record 2D correlation maps of very large macromolecular assemblies.

## Discussion

By developing a DNN to reconstruct the methyl ^1^H–^13^C correlation maps of both small and large proteins from off-resonance datasets obtained by single-pulse experiments, we have shown that the single-pulse experiment is not limited to small molecules but indeed, with the aid of deep learning it can provide high-resolution ^1^H–^13^C correlation maps of large protein complexes. In this proof-of-principle study we made use of ^13^CHD_2_ labeled proteins and obtained final ^1^H–^13^C correlation maps that are similar to standard HMQC (& ddHMQC) 2D correlation maps. However, the single-pulse approach has some shortcomings compared to the conventional FT based approaches, such as the HMQC. In a conventional experiment all points in the indirect dimension contribute to the final signal in the frequency-domain spectrum (45) so long as the evolution time is not long compared to the transverse relaxation time $T_{2}$. In contrast, in the single-pulse experiment presented here, only data recorded with ^13^C offsets, $\varpi_{RF}$ close to the chemical shift of the ^13^C nucleus, $\varpi_{C}$, contribute to the signal. Moreover, similar to other nonlinear processing strategies, the transformation performed by the developed DNN, from single-pulse off-resonance datasets to 2D correlation maps, is not linear and therefore it does not transform Gaussian noise into Gaussian noise. Consequently, (small) features that might appear in the reconstructed spectrum must not be accidentally interpreted (*SI Appendix*, Fig. S6).

As this is a proof of principle study to show that the single-pulse/DNN strategy can generate 2D correlation maps, there is room for improvement and innovation. Multifrequency ^13^C irradiation during ^1^H acquisition can be used to accelerate data acquisition (46–48). Here, a relatively shallow and simple DNN was used because it allowed for fast training and for exploration of initial conditions. The limited capacity of the relatively small DNN used here can lead to higher uncertainties in the reconstructions and larger/advanced DNNs are expected to perform better (*SI Appendix*, Fig. S7). While we have estimated the uncertainties in the reconstructed maps using the Monte Carlo dropout procedure (*Materials and Methods* and *SI Appendix*, Fig. S3) (49) it is imperative for future applications to predict quantitative uncertainties as it will allow one to identify artifacts in the reconstructions. Larger architectures will result in both robust reconstructions over a broader range of input data and quantitative estimates of uncertainties (25, 50), potentially at the risk of being harder to train. Having made the design choice of using a pair of $B_{1}$ fields whose ratio is fixed at 1:2 to record the off-resonance datasets (200 ^13^C offsets), we subsequently determined via a grid search that a reasonable pair of $B_{1}$ fields is 110/220 Hz (*SI Appendix*, Fig. S8). However, an optimum set of $B_{1}$ fields and ^13^C offsets at which ^13^C CW decoupling is carried out, without any design constraints, has not been determined. Optimizing these parameters, potentially aligned to the samples investigated and labeling schemes used, will likely improve the sensitivity and resolution of the resulting reconstructed 2D ^1^H–^13^C correlation map. It may also be fruitful to train DNNs that use regular HMQC/HSQC and off-resonance datasets to generate 2D correlation maps with higher resolution.

The single-pulse/DNN strategy was demonstrated here only for ^13^CHD_2_ methyl groups but the demonstration paves the way for applications encompassing other sites, such as more complicated spin-systems including ^13^CH_3_ methyl groups and aromatic groups in uniformly ^13^C enriched proteins and nucleic acids. One can also envisage the single-pulse/DNN strategy being extended to three-dimensional (3D) NMR experiments. In conclusion, this study not only illustrates the tremendous potential of the single-pulse/DNN strategy to obtain 2D NMR spectra from experiments without INEPT transfer periods, but it more generally again illustrates the tremendous potential of developing new NMR experiments together with customized DNNs. Specifically, the presented single-pulse/DNN strategy further adds to the emerging picture that optimal information can be extracted by developing DNNs to reconstruct readily interpretable NMR spectra from complex data that contain a large amount of information.

## Materials and Methods

### NMR Samples.

The FF domain, T4L, $\alpha_{7}\alpha_{7}$ half-proteasome and L_8_S_8_ Rubisco were all expressed in *Escherichia coli* BL21(DE3) cells grown in 100% D_2_O M9 -media containing 1 g/L ^15^NH_4_Cl, and 3 g/L [U-^2^H] glucose. Appropriate precursors were added an hour before induction as detailed elsewhere (39). The FF domain (51), T4L (52, 53), $\alpha_{7}\alpha_{7}$ half-proteasome (32), and L_8_S_8_ Rubisco (33) samples were purified as described previously. The FF domain sample contained ~1 mM [U-^15^N, ^2^H], Ile δ1-(^13^CHD_2_), Leu, Val- (^13^CHD_2_,^12^CD_3_) protein dissolved in ~550 µL 50 mM sodium acetate, 100 mM NaCl, 2 mM EDTA, 2 mM NaN_3_, pH 5.7, 100% D_2_O buffer. The T4L sample (~550 µL) contained ~1 mM (U-^15^N, ^2^H), Ile δ1-(^13^CHD_2_), Leu, Val- (^13^CHD_2_,^12^CD_3_) protein dissolved in 50 mM sodium phosphate, 25 mM NaCl, 2 mM NaN_3_, 2 mM EDTA, pH 5.5, 100% D_2_O buffer. The $\alpha_{7}\alpha_{7}$ half-proteasome sample contained ~0.5 mM (U-^15^N, ^2^H), Ile δ1-(^13^CHD_2_) of the α-monomer dissolved in ~550 µL of 20 mM sodium phosphate, 50 mM NaCl, 1 mM DTT, 1 mM EDTA, pH 6.8 100% D_2_O buffer. The L_8_S_8_ Rubisco sample contained ~0.45 mM [U-^15^N, ^2^H], Ile δ1-[^13^CHD_2_] of the large subunit monomer in complex with [U-^15^N, ^2^H] equal amounts of the small subunit monomers dissolved in ~165 µL of 50 mM Tris, 50 mM NaCl, 0.02% NaN_3,_ pH 8.0 100% D_2_O buffer (3 mm NMR tube).

### NMR Experiments.

All the NMR experiments on the FF (6.5 °C), T4L (6.5 °C), $\alpha_{7}\alpha_{7}$ (10, 50 °C), and L_8_S_8_ Rubisco (10 °C) samples were performed on 16.4 T (700 MHz) Bruker Avance III HD spectrometers equipped with TCI cryoprobes. See *SI Appendix*, Fig. S1 for additional details and the exact pulse sequence used to carry out the single-pulse experiment on ^13^CHD_2_ methyl groups. Complete off-resonances datasets at two $B_{1}$ values were recorded in ~1 (FF, T4L: 4 scans, interscan delay of 2 s), ~1.5 ($\alpha_{7}\alpha_{7}$: 8 scans, interscan delay of 1.5 s) or ~19 h (L_8_S_8_ Rubisco: 72 scans; interscan delay of 2 s).

### NMR Data Analysis.

NMRPipe (54) was used to process the NMR data and nmrglue (55) was used to transform the data between different formats. Keras (56) and Tensorflow (57) were used to both train the DNN and to construct ^1^H–^13^C correlation maps from the off-resonance datasets using the trained DNN. SPARKY (58, 59) was used to visualize the data. The quadratic interpolation implemented within SPARKY was used to determine the peak positions and intensities. PINT (60) was used to fit Gaussian shaped peaks to DNN reconstructed spectra to obtain peak intensities in the presence of peak overlap (Fig. 3 and *SI Appendix*, Fig. S4).

### Generating Synthetic Training Data.

Training data consisted of off-resonance datasets (array size: 512 × 200 × 2) as the input and the desired ^1^H–^13^C correlation maps (array size: 512 × 200) as the target. Both the off-resonance datasets and ^1^H–^13^C correlation maps consisted of 512 points in the ^1^H dimension and 200 points in the ^13^C dimension. The off-resonance datasets were calculated for two different *B_1_* values (~220, ~110 Hz) by propagating the Bloch equations for a two-spin system. The Liouvillian (61) constructed using eight basis set elements ($H_{x}$, $H_{y}$, $2H_{x}C_{x}$, $2H_{y}C_{x}$, $2H_{x}C_{y}$, $2H_{y}C_{y}$, $2H_{x}C_{z}$, $2H_{y}C_{z}$) is given by:

[1] $${\hat{L}}_{i}=-\left[\begin{array}{cccccccc} R_{2,H}^{i} & 2\pi \Omega_{H}^{i} & 0 & 0 & 0 & 0 & 0 & \pi^{1}J_{\mathrm{HC}}^{i}\\ -2\pi \Omega_{H}^{i} & R_{2,H}^{i} & 0 & 0 & 0 & 0 & -\pi^{1}J_{\mathrm{HC}}^{i} & 0\\ 0 & 0 & R_{2,MQ}^{i} & 2\pi \Omega_{H}^{i} & 2\pi \Omega_{C}^{i} & 0 & 0 & 0\\ 0 & 0 & -2\pi \Omega_{H}^{i} & R_{2,MQ}^{i} & 0 & 2\pi \Omega_{C}^{i} & 0 & 0\\ 0 & 0 & -2\pi \Omega_{C}^{i} & 0 & R_{2,MQ}^{i} & 2\pi \Omega_{H}^{i} & 2\pi B_{1} & 0\\ 0 & 0 & 0 & -2\pi \Omega_{C}^{i} & -2\pi \Omega_{H}^{i} & R_{2,MQ}^{i} & 0 & 2\pi B_{1}\\ 0 & \pi^{1}J_{\mathrm{HC}}^{i} & 0 & 0 & -2\pi B_{1} & 0 & R_{2,APH}^{i} & 2\pi \Omega_{H}^{i}\\ -\pi^{1}J_{\mathrm{HC}}^{i} & 0 & 0 & 0 & 0 & -2\pi B_{1} & -2\pi \Omega_{H}^{i} & R_{2,APH}^{i} \end{array}\right].$$

Here, ${\hat{L}}_{i}$ is the Liouvillian for ^1^H–^13^C spin-system *i*, leading to cross-peak *i*. Here, $\Omega_{H}^{i}$ is the offset of the resonance frequency (Hz) of the ^1^H nucleus of peak *i* from the ^1^H carrier, ^1^$J_{\mathrm{HC}}^{i}$ is the ^1^*J* coupling constant between the ^1^H and ^13^C nuclei of spin-system *i*, $B_{1}$ (Hz) is the strength of the ^13^C decoupling field being applied along x, $\Omega_{C}^{i}$ is the offset of the resonance frequency (Hz) of the ^13^C nucleus of *i* from the ^13^C carrier, $R_{2,H}^{i}$ is transverse relaxation rate of the ^1^H nucleus, $R_{2,MQ}^{i}$ describes the relaxation of the multiquantum terms ($2H_{j}C_{k}$, $j,k\in \left(x,y\right)$) of *i* and $R_{2,APH}^{i}$ describes the relaxation of antiphase terms $2H_{x}C_{z}$ and $2H_{y}C_{z}$. Cross-correlated relaxation, which could lead to cross-relaxation between the different terms, was not considered. The FID originating from spin-system *i* is given by, $s_{i}\left(t\right)={\vec{D}e}^{-{\hat{L}}_{i}t}{\vec{V}}_{i}\left(0\right)$ with ${\vec{V}}_{i}\left(0\right)={\left(I_{0}^{i},0,0,0,0,0,0,0\right)}^{T}$ and $\vec{D}=\left(1,\sqrt{-1},0,0,0,0,0,0\right)$. $I_{0}^{i}$ is the starting magnetization originating from *i*. The coupled (reference) FID, $s_{u,i}\left(t\right)$ is similarly calculated with $B_{1}$ set to 0 in the above expression for ${\hat{L}}_{i}$. It is worth noting that the Liouvillian (Eq. **1**) does not depend on the single-quantum ^13^C transverse relaxation rate ($R_{2,C}^{i}$) that describes the relaxation of terms like $C_{x}$ and $C_{y}$. Specifically, in the limiting case where ^13^C saturation is applied far off-resonance, the ^1^H signal is a doublet with splitting of ^1^*J*_HC_ and the linewidth is ${∼R}_{2,H}$/π (**SI Appendix*, SI Text*). In the other extreme, which is when the ^13^C saturation is on-resonance, the ^1^H signal is a singlet with an effective transverse relaxation rate of ~$R_{2,H}-\frac{{R_{2,H}-R}_{2,MQ}}{1+{\left(2B_{1}/J_{HC}\right)}^{2}}$ (**SI Appendix*, SI Text*). Hence, the off-resonance datasets do not contain information on $R_{2,C}$ that determines the linewidth in HSQC type spectra and only contains limited information on $R_{2,MQ}$, which relate to the linewidth in HMQC type spectra. In order to train the neural network to perform robust transformations, and since the input dataset contains only very limited information on the transverse (multiquantum) ^13^C relaxation rate, it was decided to have a target with a fixed linewidth in the ^13^C dimension. It should be stressed, however, that the simulations of the input dataset are accurate and robust (Eq. **1**) and they do take such, albeit small, dependences in the input data into account. Also, fixing the linewidth in the ^13^C dimension rather than relating it to $R_{2,MQ}$ improves the quality of the reconstructed ^1^H–^13^C correlation maps when the $R_{2,MQ}$ values are very large.

To generate random off-resonance datasets for training and evaluation, experimental parameters ($B_{0}$, $B_{1}$, ^13^C sweepwidth, ^1^H sweepwidth, and phase of the FID) are randomly chosen over the ranges given in *SI Appendix*, Table S1. The number of peaks (Npeaks) per spectrum is set to a random number between 1 and Npeaksmax (the maximum number of peaks in the spectrum). For each of the Npeaks peaks, peak specific parameters (ϖHi,ϖCi,J1HCi,R2,Hi,R2,MQi,R2,APHi, and $I_{0}^{i}$) are randomly chosen over the ranges specified in *SI Appendix*, Table S1. $\varpi_{H}^{i}$ and $\varpi_{C}^{i}$ are respectively the chemical shifts (ppm) of the ^1^H and ^13^C nuclei of peak *i*. For a given ^13^C offset the peak (*i*) specific FID ($s_{i}\left(t\right)$) is calculated for times varying from 0 to 64 ms in steps of 1/(^1^H sweepwidth) using ${\hat{L}}_{i}$ defined above. FIDs from all the peaks are summed to obtain the sample’s difference FID, $s_{D}\left(t\right)=\sum_{i=0}^{\mathrm{Npeaks}}\left(s_{i}\left(t\right)-s_{u,i}\left(t\right)\right)$. The sample FID is cosine square apodised, zero filled to 512 points and Fourier transformed to obtain the ^1^H spectrum while decoupling at a specific ^13^C offset using a specific $B_{1}$ field. This process is repeated for all the desired offsets (200) and two $B_{1}$ fields to generate the input off-resonance datasets. The off-resonance datasets were scaled so that the maximum value is unity and random Gaussian noise with varying maximum values (*SI Appendix*, Table S1) was added following which the off-resonance datasets were rescaled so that the maximum value was unity. The corresponding training target, the ^1^H–^13^C correlation map, contains Gaussian peaks at resonance positions of the peaks that were used to construct the off-resonance datasets. The linewidth of peaks in the ^1^H dimension is determined by $R_{2,H}$ (with the minimum linewidth set to 20 Hz). On the other hand, the linewidth of peaks in the ^13^C dimension is fixed to 30 Hz. The intensity of the peaks is determined by the peak specific $I_{0}$ values. Noise was not added to the training target.

### Training the DNN.

The DNN used to transform the ^1^H–^13^C difference off-resonance datasets into the standard ^1^H–^13^C correlation is a convolutional neural network (62) with skip connections (63) that contained seven hidden layers (~4.5 million weights; *SI Appendix*, Fig. S2). The DNN was trained to transform the synthetic input off-resonance datasets ($B_{1}$ ~220, ~110 Hz) into the desired output ^1^H–^13^C correlation maps using backpropagation via the ADAM optimization algorithm (64). The loss function was the MSE between the intensities of the predicted and desired noise-free training target ^1^H–^13^C correlation maps. To reduce the chances of overfitting, dropout (30%) (65) and L2 regularization were also used during training. At the start of training Npeaksmax was set to 10 and was increased to a maximum value of 1,500 over a few rounds of training (*SI Appendix*, Figs. S2 and S3). The DNN was trained using 475,000 training spectra containing a total of ~148 million peaks (*SI Appendix*, Fig. S2).

### Reconstructing the ^1^H–^13^C Correlation Maps from Off-Resonance Datasets Using the DNN.

The experimental off-resonance ($B_{1}$ ~220, ~110 Hz) datasets recorded at 200 ^13^C decoupling offsets that cover the entire methyl ^13^C chemical shift region (~30 ppm ~5,300 Hz; 16.4 T; See *SI Appendix*, Table S1) and the reference ^1^H 1D spectrum were manipulated using NMRPipe to construct the difference off-resonance spectra (^1^H sweep width of ~5 ppm) of the appropriate size (512, 200). The ^1^H FIDs were phased, cosine square apodized, and appropriately zero filled as was done during training and so that the final spectrum has 512 points in the ^1^H dimension. Subsequently the data were Fourier transformed to generate the difference off-resonance spectra that were appropriately scaled, with a maximum value of 1, and used as the inputs to the DNN to construct the ^1^H–^13^C correlation map. Twenty ^1^H–^13^C correlation maps were predicted via a Monte Carlo dropout procedure (49) with 30% dropout in all but the last hidden layers (*SI Appendix*, Figs. S2 and S3). The mean of the 20 predicted $I\left(\varpi_{H},\varpi_{C}\right)$ maps was used as the reconstructed ^1^H–^13^C correlation map and as described in *SI Appendix*, Fig. S3 the SD, $\sigma_{I_{\mathrm{Pred}}}$, among the 20 predicted $I\left(\varpi_{H},\varpi_{C}\right)$ maps was used to estimate the point-by-point uncertainty, $\sigma_{\mathrm{Recon}}$, in the reconstructed correlation map with $\sigma_{\mathrm{Recon}}$ = 1.4 $\sigma_{I_{\mathrm{Pred}}}$. Peak intensities and related uncertainties were then obtained either directly from the mean (of 20) $I\left(\varpi_{H},\varpi_{C}\right)$ and the $\sigma_{\mathrm{Recon}}\left(\varpi_{H},\varpi_{C}\right)$ maps using SPARKY or in the case of overlap from the 20 predicted $I\left(\varpi_{H},\varpi_{C}\right)$ maps using PINT to fit Gaussian functions at the desired peak positions. The uncertainty in the fitted peak intensity was assumed to be 1.4 times the SD in the 20 intensities.

## Supplementary Material

Appendix 01 (PDF)

## Data Availability

Keras/TF scripts to carry out the reconstruction along with weights and examples are available online (https://github.com/hansenlab-ucl/SinglePulse13CH) (66). Scripts are available both for processing within Bruker TopSpin on the NMR spectrometer and for processing off the spectrometer. All other data are included in the manuscript and/or *SI Appendix*.
