# Supplementary material for: Two-dimensional NMR from a single pulse: Reconstructing heteronuclear 2D spectra via off-resonance decoupling and deep neural networks
Source: Proc Natl Acad Sci U S A. 2026 Apr 21;123(17):e2527937123. doi: 10.1073/pnas.2527937123 (PMC13123836; doi:10.1073/pnas.2527937123)
Supplement: Supplementary file 1 — Appendix 01 (PDF) [file pnas.2527937123.sapp.pdf]

Supporting Information

Two-dimensional NMR from a Single Pulse: Reconstructing Heteronuclear 2D spectra via off-resonance decoupling and Deep Neural Networks

SI Text

^{1}H spectrum in the presence of CW ^{13}C decoupling.

To obtain an intuitive insight into the signals observed in the single-pulse experiment, it is of interest to understand the ^{1}H spectrum in the presence of on-resonance ^{13}C decoupling. Without any loss of generality, we consider a ^{1}H-^{13}C spin system, where the ^{1}H and ^{13}C carriers are placed on the ^{1}H and ^{13}C chemical shifts, respectively, where the Hamiltonian is $2\pi\ ^{1}J_{HC}H_zC_z + 2\pi B_1 C_x$ as $\Omega_H, \Omega_C\ = 0$ rad/s. For simplicity, we start by considering the case when there is no decoupling (B_1 = 0 Hz) equivalent to the case when B_1 is applied far off-resonance. To understand how transverse, H_x, magnetization evolves we can restrict ourselves to a basis containing two elements, H_x, $2H_yC_z$, which evolve according to the 2×2 Liouvillian ($\hat{L}_{2\times2}$) given by:

$$\hat{L}_{2\times2} = -\begin{bmatrix} R_{2,H} & \pi\ ^{1}J_{HC} \\ -\pi\ ^{1}J_{HC} & R_{2,APH} \end{bmatrix} \qquad \text{Eq S1}$$

$\hat{L}_{2\times2}$ is derived from the 8×8 Liouvillian given in the text (Eq 1). The eigenvalues of $\hat{L}_{2\times2}$ that report on the frequency and linewidths of the signals observed are trivially are given by:

$$\lambda_{\pm} = -\frac{\left(R_{2,H}+R_{2,APH}\right)}{2} \pm \pi i\ ^{1}J_{HC}\sqrt{1-\left(\frac{R_{2,APH}-R_{2,H}}{2\pi\ ^{1}J_{HC}}\right)^2} \qquad \text{Eq S2}$$

For the cases studied here, $2\pi\ ^{1}J_{HC} \gg \left|R_{2,H} - R_{2,APH}\right| \Rightarrow \left|\frac{R_{2,APH}-R_{2,H}}{2\pi\ ^{1}J_{HC}}\right| \ll 1$. Thus, the spectrum consists of a doublet with a splitting slightly less than $^{1}J_{HC}$, where the width of each of the peaks is determined by a relaxation rate $\left(R_{2,H} + R_{2,APH}\right)/2$. It is often assumed that $R_{2,APH} \approx R_{2,H} + R_{1,C}$, in which case the width of each of the peaks is determined by the effective relaxation rate $R_{2,H} + R_{1,C}/2$. For the ^{13}CHD$_2$ groups under investigation here the longitudinal ^{13}C relaxations, $R_{1,C}$, are rather slow and on the order of 0.5 – 0.1 s^{-1}, so that $R_{2,APH} \approx R_{2,H}$ and the width of the peaks in the doublet is essentially determined by $R_{2,H}$.

Next, we consider the case when on-resonance ^{13}C decoupling is carried out restricting ourselves to the case when $B_1 > 0$. The relevant basis set consists of three terms, H_x, $2H_yC_y$, $2H_yC_z$, whose evolution assuming $R_{2,APH} = R_{2,H}$ is described by the 3×3 Liouvillian ($\hat{L}_{3\times3}$):

$$\hat{L}_{3\times3} = -\begin{bmatrix} R_{2,H} & 0 & \pi\ ^{1}J_{HC} \\ 0 & R_{2,MQ} & 2\pi B_1 \\ -\pi\ ^{1}J_{HC} & -2\pi B_1 & R_{2,H} \end{bmatrix} \qquad \text{Eq S3}$$

Under the simplifying assumption that $R_{2,H} = R_{2,MQ} = R_2$, the eigenvalues of $\hat{L}_{3\times3}$ are:

$$\left\{-R_2,\ -R_2 \pm i2\pi B_1\sqrt{1+\left(\frac{{}^1J_{HC}}{2B_1}\right)^2}\right\} \qquad \text{Eq S4}$$

The first eigenvalue gives rise to the decoupled central peak, while the second and third eigenvalues give rise to decoupling sidebands at offsets of $\pm B_1\sqrt{1+\left({}^1J_{HC}/2B_1\right)^2}$ (Hz). The detected signal $s(t)$ is given by $\vec{D}e^{-\hat{L}_{3\times3}t}\vec{V}(0)$ with $\vec{D} = \vec{V}(0) = (1,0,0)$ which is $\left[\frac{4B_1^2}{{}^1J_{HC}^2+4B_1^2} + \frac{{}^1J_{HC}^2\cos\left(2\pi B_1 t\sqrt{1+\left({}^1J_{HC}/2B_1\right)^2}\right)}{\left({}^1J_{HC}^2+4B_1^2\right)}\right]e^{-R_2t}$ showing that the size of the first term that gives rise to the decoupled signal increases with B_1. On the other hand, increasing B_1decreases the size of the sidebands and moves them away from the central peak.

Without the simplifying assumption that $R_{2,H} = R_{2,MQ}$, the eigenvalues of $\hat{L}_{3\times3}$ are complicated expressions and we instead obtained an approximate solution. The ^{13}C spins experience an effective field arising due to the B_1 applied along the *x* axis and the ${}^1J_{HC}$ coupling. The cosine of the angle θ between the effective axis and the *x* axis is given by $\sqrt{4\pi^2B_1^2/\left(4\pi^2B_1^2+\pi^2\ {}^1J_{HC}^2\right)}$. Here, the $2\pi B_1$ (rad/s) arises due to the ^{13}C decoupling field applied along the *x* axis. $\pm\pi\ {}^1J_{HC}$ (rad/s) is the offset between the chemical shift of the ^{1}H nucleus and the precession frequencies of the two components of the ^{1}H doublet. The effective relaxation rate of the component locked along the effective field is given by ~ $R_{2,H}\cos^2(\theta) + R_{2,MQ}\sin^2(\theta) = R_{2,H} - \left(R_{2,H} - R_{2,MQ}\right)\Big/\left(1+\left(\frac{2B_1}{{}^1J_{HC}}\right)^2\right)$ showing that the relaxation rate of the decoupled peak has a small dependence on $R_{2,MQ}$, as $\left|2B_1/\ {}^1J_{HC}\right| > 1$. The sidebands arise due to precession around the effective field and the relaxation rate is $\left(R_{2,H} + R_{2,MQ}\right)/2$.

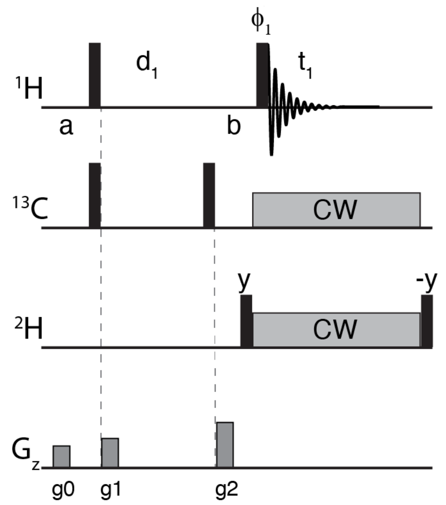

Fig. S1. 'Single Pulse' NMR experiment for recording 2D ^1H-^{13}C correlation maps from $^{13}CHD_2$ enriched protein samples. The pulses and gradients between points a and b are applied to destroy any unwanted coherences. Narrow black bars denote $\pi/2$ pulses and are of x phase unless indicated. ^1H and ^{13}C $\pi/2$ pulses are applied at the highest possible power while the ^2H $\pi/2$ pulses were ~150 μs. The ^1H carrier is placed on water, the ^2H carrier is placed at ~0.5 ppm in the middle of the methyl region. 0.5 kHz CW ^2H decoupling is carried out during ^1H detection. At point a, the ^{13}C carrier is placed at ~18.5 ppm in the middle of the methyl region and moved to the desired offset at point b. During detection, CW ^{13}C decoupling was carried out with B_1 values of ~220 or ~110 Hz. ϕ_1, ϕ_{rec} = x, y, -x, -y. The ^1H FID was acquired for 64 ms (t_1). The gradients g0, g1 and g2 were applied for a duration of 300 μs with relative strengths of 0.35, 0.5 and 1.0 respectively. The experiment is carried out in a pseudo 2D manner with the ^{13}C CW decoupling carried out at different offsets. At 16.4 T (700 MHz) data was collected for a total of 200 ^{13}C offsets between -2636.75 and +2636.75 Hz around the reference carrier position (~18.5 ppm). A 1D ^1H spectrum without ^{13}C CW decoupling is also recorded. All the data was recorded with the ^1H sweep width set to ~16 ppm.

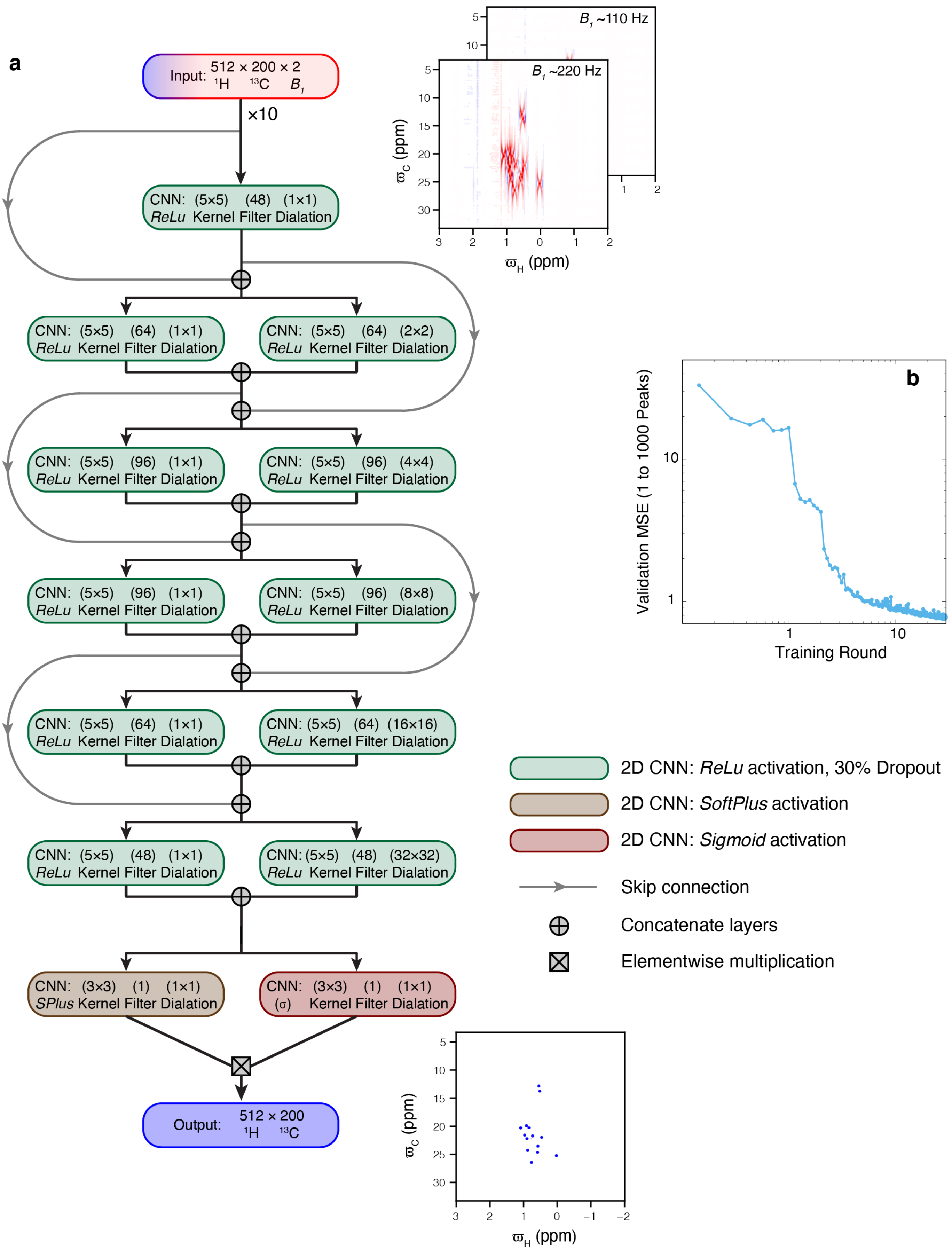

Fig. S2. a) The DNN used in this study to convert the ^{1}H-^{13}C off-resonance data into a standard ^{1}H-^{13}C correlation map consists of seven hidden layers (4542514 ~4.5 million weights). All the hidden layers are comprised of two dimensional convolutional neural networks (2D CNNs) whose parameters are indicated in boxes. Except for the last hidden layer, the activation function is the rectified linear unit (*ReLu*). The softplus (*SPlus*) and sigmoid (σ) activation

functions are used in the last hidden layer. The grey lines with arrows denote skip connections. The + in the circle indicates concatenation while the × in a square box indicates elementwise multiplication. The input consists of two (B_1 = ~220, ~110 Hz) difference off-resonance datasets each of size 512×200. The input datasets are normalized by dividing the datasets by the largest intensity in both planes and internally multiplied by 10 (as indicated above) before being passed on to the first hidden layer. Padding is carried out so that the size of the output from every layer is 512×200 along the first two axis. Dropout (30%) was applied to all the hidden layers other than the last ones. Dropout was used both during training and prediction (inference). In the dropout procedure a node is deleted (by turning off all its connections) with a probability termed the dropout rate (30% here). While training the DNN, application of dropout reduces overfitting by preventing co-adaptation of nodes. b) Plot showing that the validation mean square error (MSE) decreases as training proceeds. As mentioned in the materials and methods section, the number of peaks (Npeaks) per spectrum is set to a random number between 1 and Npeaksmax (the maximum number of peaks in the spectrum). The validation dataset consists of 5000 difference ^{1}H-^{13}C off-resonance datasets and the desired output 2D ^{1}H-^{13}C correlation maps with Npeaksmax set to 1000. The validation dataset is not used during training and the weights at various stages during training were used to predict 2D ^{1}H-^{13}C correlation maps from the validation difference ^{1}H-^{13}C off-resonance datasets. The differences in the (point by point) intensities between the predicted 2D ^{1}H-^{13}C correlation maps and the desired validation output 2D ^{1}H-^{13}C correlation maps was used to calculate the validation MSE. 95,000 training spectra were used during each round of training. Initially training was carried out using simple spectra with Npeaksmax set to 10 in the first round. More complex spectra were used during the subsequent rounds of training with Npeaksmax for the second, third, fourth and fifth rounds of training set to 100, 500, 1000 and 1500 respectively. Spectra were not reused during the first five rounds of training. After the fifth round of training, training data from rounds four (1 to 1000 peaks; Npeaksmax=1000) and five (1 to 1500 peaks, Npeaksmax=1500) was reused to carryout training for a total of 30 rounds. Thus, the total size of the training dataset was 475,000 spectra that contained ~147.5 million peaks.

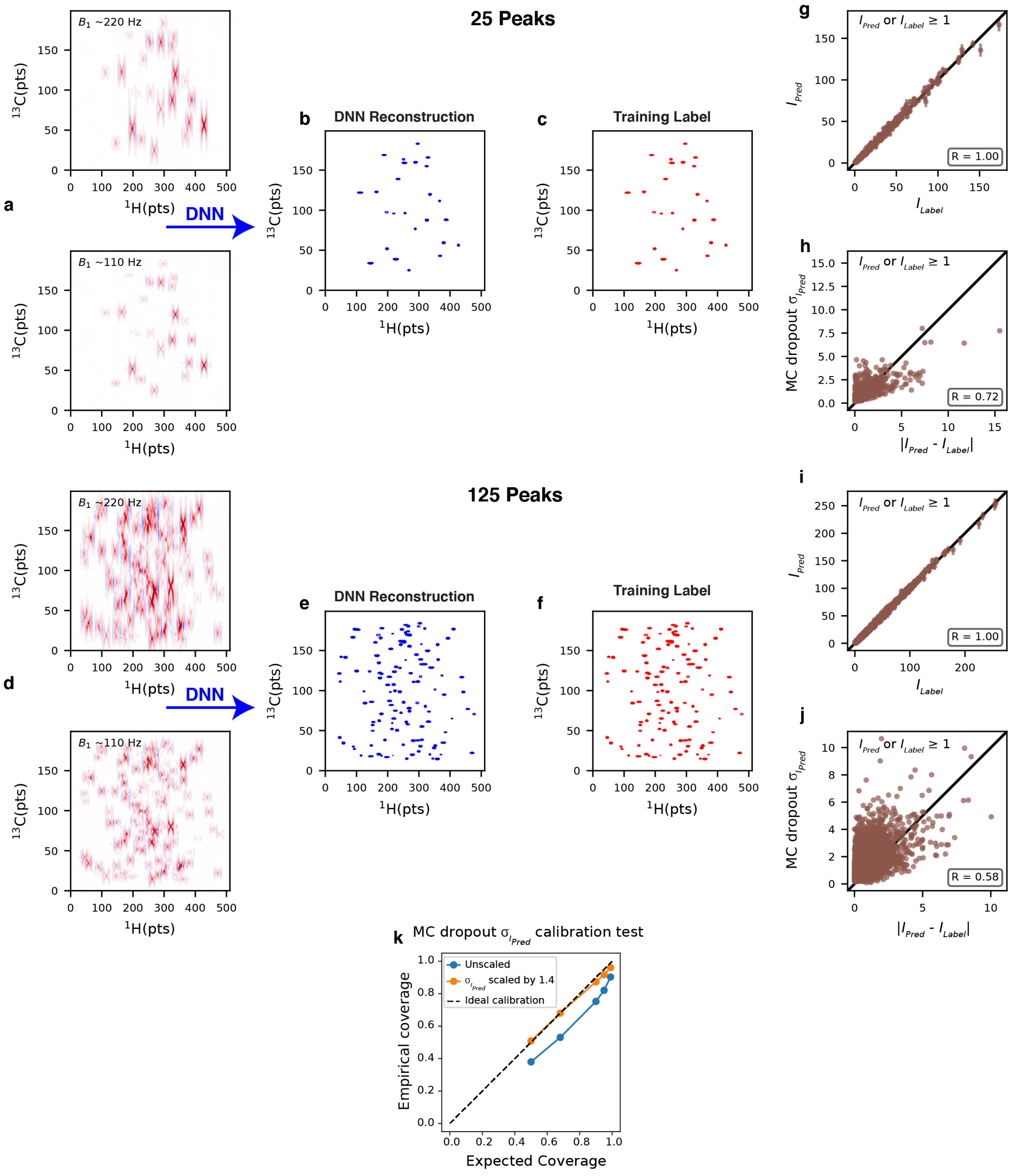

Fig. S3. Evaluating the DNN from Fig. S2 using simulated data. The DNN converts simulated difference off-resonance datasets with 25 (a) and 125 (d) correlations into 2D ^{1}H-^{13}C correlation maps (b,e). For comparison the desired outputs (training label data) are shown in c (25 peaks) and d (125 peaks). A Monte Carlo dropout procedure with 20 trials was used obtain the reconstructed ^{1}H-^{13}C correlation maps. As mentioned in Fig. S2, by deleting nodes probabilistically at the dropout rate (30% here), the dropout procedure reduces overfitting during training by preventing co-adaptation of nodes. In the Monte Carlo dropout procedure, dropout is applied at inference also, while the trained DNN is used to predict the output 2D ^{1}H-^{13}C correlation map N times (N = 20 here) for a given input off-resonance dataset. This results in N slightly different output 2D ^{1}H-^{13}C correlation maps that arise from (an ensemble of) N networks with slightly different graph structures (due to dropout). The predicted ($I_{Pred}(\varpi_H, \varpi_C)$) output 2D ^{1}H-^{13}C correlation map is the point-by-point mean of the ensemble of the N (=20) predictions while the point-by-point

standard deviation ($\sigma_{I_{Pred}}(\varpi_H, \varpi_C)$) is related to the uncertainty in the prediction though it needs to be calibrated as described below. (g,i) Comparison of the intensities (I_{Pred}) at various points of the DNN predicted spectra with intensities (I_{Label}) from the training label. In g (i) each point in b (e) is compared to the corresponding point in c (f). Comparison of (h,j) $\sigma_{I_{Pred}}$ with $|I_{Pred} - I_{Label}|$. The positive correlation suggests that the $\sigma_{I_{Pred}}$ values are meaningful estimates of the point-by-point intensity uncertainty (σ_{Recon}) in the reconstructed map. (k) Calibration test for the $\sigma_{I_{Pred}}$ values. If the $\sigma_{I_{Pred}}$ values are perfect estimates of the uncertainty, we expect ~68% of the $|I_{Pred} - I_{Label}|$ values to be ≤ $\sigma_{I_{Pred}}$, ~95% of the $|I_{Pred} - I_{Label}|$ values to be ≤ 2$\sigma_{I_{Pred}}$ and so on. The graph in k plots the fraction of $|I_{Pred} - I_{Label}|$ values empirically determined (empirical coverage) to lie within the expected value based on the $\sigma_{I_{Pred}}$ values versus the expected fraction (expected coverage). It is clear that the fraction of $|I_{Pred} - I_{Label}|$ values that lie within the expected range is reasonable but slightly underestimated based on the $\sigma_{I_{Pred}}$ values (compare blue and black curves). However, ~68% of the $|I_{Pred} - I_{Label}|$ values lie within 1.4$\sigma_{I_{Pred}}$ and recalculating curve (orange) with scaled $\sigma_{I_{Pred}}$ leads to an excellent agreement (orange vs black) showing that the dropout MC derived $\sigma_{I_{Pred}}$ values are reasonable and that 1.4$\sigma_{I_{Pred}}$ is a good estimate of the uncertainty (σ_{Recon}) in the reconstructed maps. Thus, in this study 1.4$\sigma_{I_{Pred}}$ is used as an estimate of the uncertainty (σ_{Recon}) in the intensities of the DNN derived ^{1}H-^{13}C correlation maps. The plot shown in (k) was calculated using 250 difference off-resonance datasets, the corresponding ^{1}H-^{13}C correlation maps and DNN reconstructions. Each of the 250 datasets contained a random number of peaks between 1 and 1000. In g-k only points in the correlation maps with I_{Pred} or $I_{Label} > 1$ are considered.

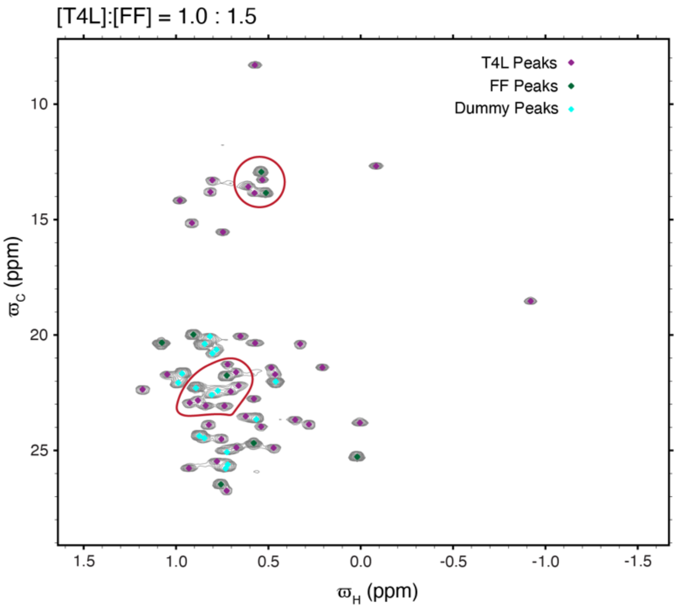

Fig. S4. T4L and FF peaks for which intensities were obtained by Gaussian peak shape fitting are shown in purple and green respectively on a spectrum which is a sum of the T4L and FF spectra in the ratio 1:1.5. Despite the overlap (for example circled regions) peak intensities were obtained for 40 T4L and 8 FF peaks. The other correlations for which intensities were not obtained arise from the direct overlap of correlations and these too were often considered (cyan dummy peaks) while obtaining the peak intensities of the desired peaks that are in the neighborhood of the dummy peaks.

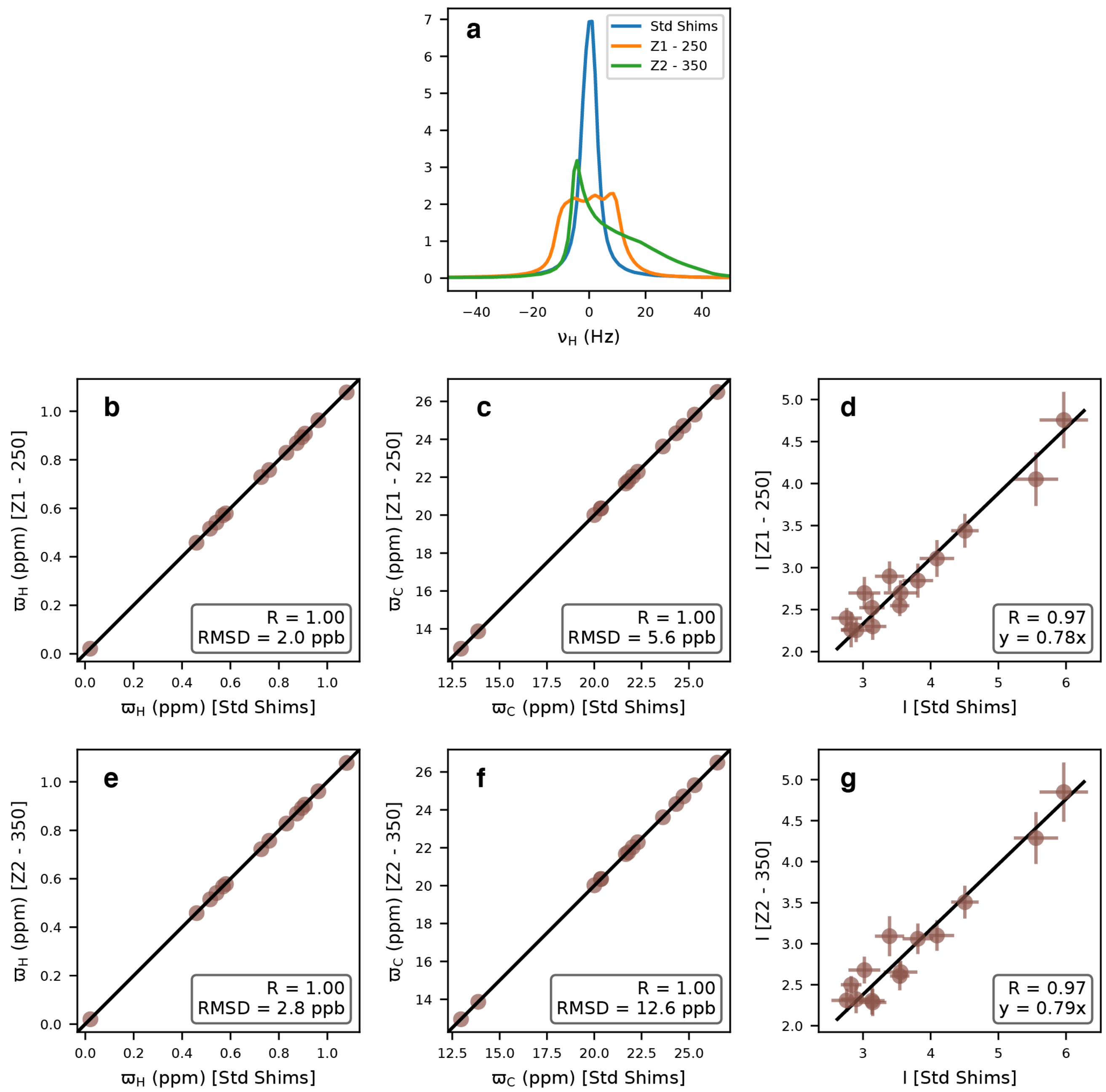

Fig. S5. Testing the robustness of the DNN by poorly shimming the magnet. a) Spectrum of the residual ^{1}H signal in the FF sample with standard shims (Std Shims), Z1 decreased by 250 units (Z1 - 250) and Z2 decreased by 350 units (Z2 - 350). Comparison of ^{1}H (b), ^{13}C (c) peak positions and peak intensities (d) between the FF domain methyl ILV ^{1}H-^{13}C maps constructed by the DNN with standard shims and with Z1 decreased by 250 units. Comparison of ^{1}H (e), ^{13}C (f) peak positions and peak intensities (g) between the FF domain methyl ILV ^{1}H-^{13}C maps constructed by the DNN with standard shims and the Z2 decreased by 350 units.

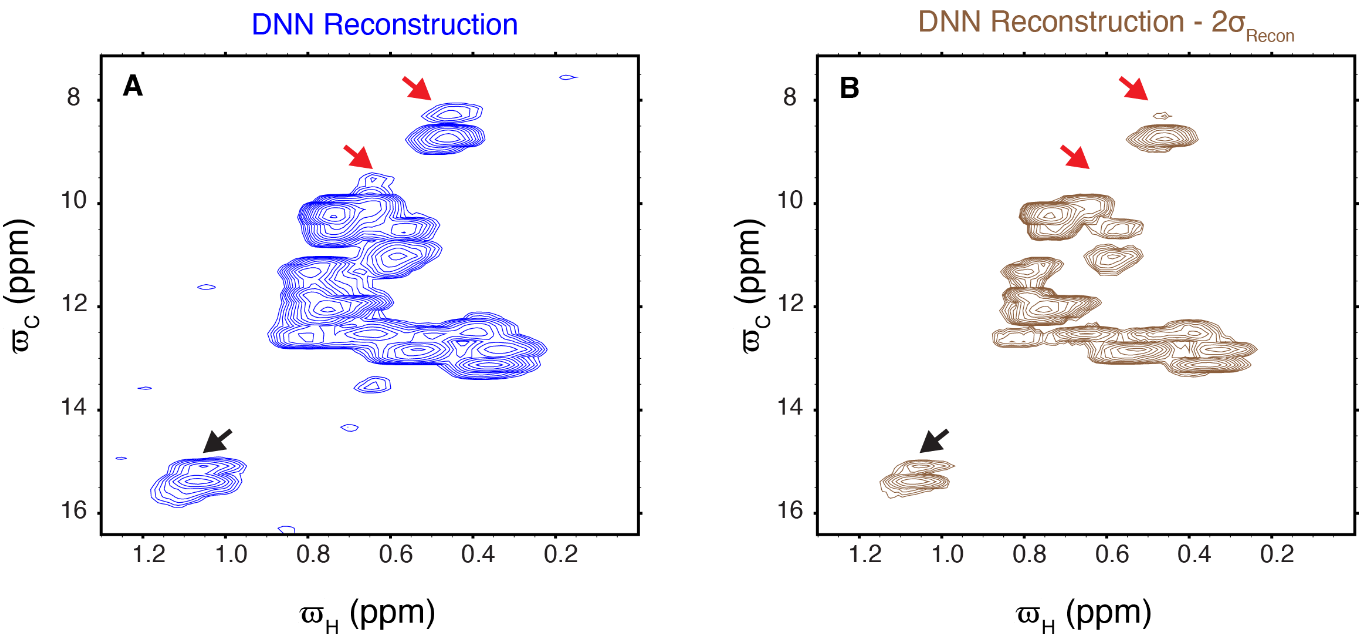

Fig. S6. Identifying unreliable features in the DNN reconstructed ^{1}H-^{13}C correlation maps of the Ile $\delta1$ ^{13}C-^{1}H correlation maps (10 °C, 16.4 T; 700 MHz) of the $\alpha_7\alpha_7$ proteasome. (a) DNN reconstruction (I_{Pred}) and (b) a ^{13}C-^{1}H correlation map ($I_{Pred} - 2\sigma_{Recon}$) that is the difference of DNN reconstruction and twice the uncertainty. Only positive contours are shown in the difference map (b). The data used here is from Fig. 4b. Peaks indicated using red arrows in the reconstruction (a) are not clearly visible in the difference map (b) suggesting that these (peaks) features in the DNN reconstruction (a) may not be reliable and should not be considered while analyzing the DNN reconstructed ^{1}H-^{13}C correlation maps. On the other hand, the small peak indicated by the black arrow in the reconstruction (a) is visible in the difference map (b) suggesting that this peak in the DNN reconstruction (a) is reliable and can be considered while analyzing the DNN reconstructed ^{1}H-^{13}C correlation maps.

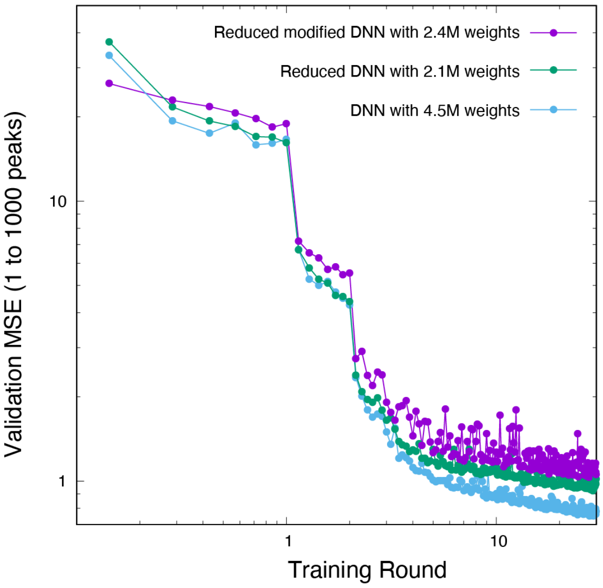

Fig. S7. The efficacy of the reconstruction procedure depends on the architecture of the DNN used to reconstruct the ^{1}H-^{13}C correlation map. The above figure compares the variation in the validation MSE for different DNNs as a function of training round. See Fig. S2 for more details. The 'reduced modified DNN' (~2.4 million weights, purple line) also consists of seven hidden layers without skip connections while the 'reduced DNN' (~2.1 million weights, green line) has the same topology as the DNN in Fig. S2 but with lesser number of weights due to a reduction in the number of filters per layer. The performance of both the reduced DNNs that have similar number of weights differs from one another and are both worse than the performance of the larger DNN used in this study (cyan line; Fig. S2) suggesting that reconstructions may be further improved by exploring other DNN architectures.

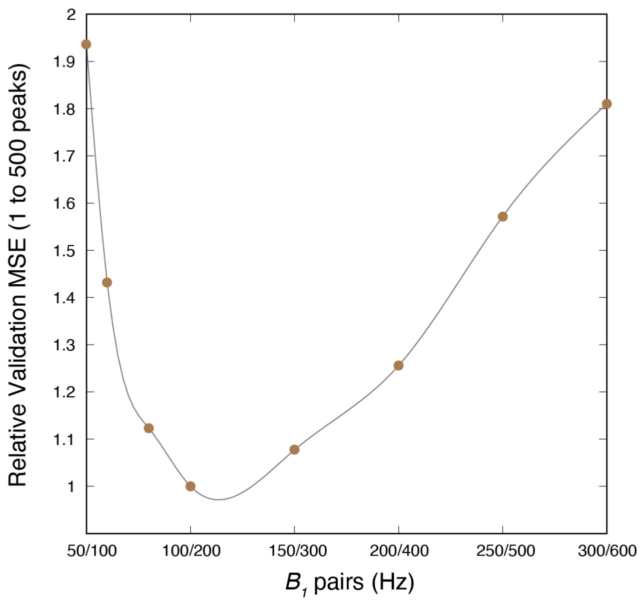

Fig. S8: The efficacy of the reconstruction procedure depends on the choice of the decoupling B_1 fields used to record off-resonance datasets. Having decided that the reconstruction procedure will use off-resonance datasets (200 ^{13}C offsets) recorded at a pair of B_1 values with a fixed ratio of 1:2 we decided to determine a reasonable B_1 pair. To determine the optimal B_1 values, training datasets (maximum of 500 peaks) were generated for eight (1:2) B_1 pairs (50/100, 60/120, 80/160, 100/200, 150/300, 200/400, 250/500 and 300/600 Hz) that were subsequently used to separately train a small DNN (~1.3 million weights) with seven hidden layers. The results of the training exercise were evaluated using a validation dataset (size 5000) that contained spectra with a random number (between 1 and 500; Npeaksmax=500) of peaks. The lowest validation MSE (0.633) was obtained for the model trained on the dataset generated with B_1 values of 100/200 Hz. Above the variation of the relative validation MSE is shown as function of the B_1 fields used to generate the training data. The relative validation MSE was obtained by dividing the validation MSE by the minimum value of 0.633. The line is drawn only to guide the eye and indicates that the validation MSE does not change very much (~10 %) when the lower B_1 value is varied from 80 to 150 Hz resulting in the choice here of 110 Hz. Various other parameters like the number of B_1 fields, their exact values, offset spacing etc. can be varied in a bigger hyperparameter search to improve the efficacy of the single-pulse approach.

Spectral Parameters		
**Parameter**	**Range**	**Comments**
B_0	± 0.5 MHz around the desired B_0	For 700 MHz: 699.5 to 700.5 MHz
Number of Offsets (Noffset)	200 [Fixed]	
Evolution time (^{1}H dimension)	64 ms [Fixed]	
Number of points (^{1}H dimension)	512 [Fixed]	
Spectrum Center (^{1}H,^{13}C)	(0 ppm, 17.5 ppm) [Fixed]	For convenience, does not matter
^{1}H sweep width	4.85 to 5.15 ppm	
^{13}C sweep width	29.0 to 31.0 ppm	
B_1 (First plane)	205 to 235 Hz	B_1 does not have to be accurately calibrated.
B_1 (Second plane)	(0.48 to 0.52)× B_1 (First plane)	
Phase error	-5° to 5°	Phase error is introduced as the spectrum cannot be perfectly phased.
Gaussian error	1%	Maximum noise in the input off-resonance datasets.
Number of Peaks (Npeaks)	1 to 1500	To include more overlapped examples during training.
**Peak Specific Parameters**		
**Parameter**	**Range**	**Comments**
^{1}H chemical shift (ppm)	1/3 peaks (-2.25 to 2.25 ppm)<br>2/3 peaks triangularly distributed around 0 between (-1.75 to 1.75 ppm)	Final range (-2.25 to 2.25 ppm)
^{13}C chemical shift (ppm)	± 13 ppm	Final range (4.5 to 30.5 ppm)
$^1J_{H,C}$	115 to 140 Hz	
$R_{1,C}$	0.25 to 10 s^{-1}	Internal parameter
$R_{2,C}$	1.25 to 125 s^{-1}	Internal parameter
$R_{1,H}$	0.25 to 15 s^{-1}	Internal parameter
$R_{2,H0}$	1.25 to 125 s^{-1}	Internal parameter
$S1$	0.1 to 1.0	Internal parameter
$S2$	0.1 to 0.75	Internal parameter
$S3$	0.5 to 10.0	Internal parameter
$R_{2,HEF}$	$S1$ ×(1.25 to 125.0)	Internal parameter
$R_{2,H}$	$R_{2,H0}$ + $R_{2,HEF}$	Restricted to: 2.5 and 250 s^{-1}
$R_{2,HE}$	$R_{2,H}$ - $R_{2,H0}$	Internal parameter, Restricted: 0 to 150 s^{-1}
$R_{2,MQ}$	$R_{2,HE} + S2 \times R_{2,C}$	Restricted to: 1.25 and 250 s^{-1}
$R_{2,APH}$	$R_{2,H} + S3 \times R_{1,C}$	
I_0	0.01 to 1.0	To account for starting intensity variations due to varying ^{1}H R_1s, sample mixtures with molecules of varying concentrations.

Table S1. Parameters used to generate training data to train the DNN for reconstructing methyl ^{1}H-^{13}C for off-resonance datasets. Unless specified '*x* to *y*' in the second column means that a uniformly distributed random real number *r* between *x* and *y* is chosen. In the third column 'Restricted to: *x* to *y*' means that if $r < x$, *r* is set to *x* and if $r > y$, *r* is set to *y*. Empirically we find that the trained DNN is able to reconstruct isolated peaks from features that have a S/N of ~3 in the off-resonance datasets.
